# Improving tumor treatment through intratumoral injection of drug-loaded magnetic nanoparticles and low-intensity ultrasound

**DOI:** 10.1038/s41598-024-52003-9

**Published:** 2024-01-16

**Authors:** Asma Hosseinpour, Madjid Soltani, Mohammad Souri

**Affiliations:** 1 Department of Mechanical Engineering, K. N. Toosi University of Technology, Tehran, Iran; 2 Department of Electrical and Computer Engineering, University of Waterloo, Waterloo, Canada; 3 Centre for Biotechnology and Bioengineering (CBB), University of Waterloo, Waterloo, Canada; 4 Department of Integrative Oncology, BC Cancer Research Institute, Vancouver, Canada; 5 Centre for Sustainable Business, International Business University, Toronto, Canada; 6 Department of NanoBiotechnology, Pasteur Institute of Iran, Tehran, Iran

**Keywords:** Biophysics, Cancer, Computational biology and bioinformatics, Medical research, Oncology, Engineering, Mathematics and computing

## Abstract

The intratumoral injection of therapeutic agents responsive to external stimuli has gained considerable interest in treating accessible tumors due to its biocompatibility and capacity to reduce side effects. For the first time, a novel approach is explored to investigate the feasibility of utilizing low-intensity ultrasound in combination with intratumoral injection of drug-loaded magnetic nanoparticles (MNPs) to thermal necrosis and chemotherapy with the objective of maximizing tumor damage while avoiding harm to surrounding healthy tissue. In this study, a mathematical framework is proposed based on a multi-compartment model to evaluate the effects of ultrasound transducer’s specifications, MNPs size and distribution, and drug release in response to the tumor microenvironment characteristics. The results indicate that while a higher injection rate may increase interstitial fluid pressure, it also simultaneously enhances the concentration of the therapeutic agent. Moreover, by increasing the power and frequency of the transducer, the acoustic pressure and intensity can be enhanced. This, in turn, increases the impact on accumulated MNPs, resulting in a rise in temperature and localized heat generation. Results have demonstrated that smaller MNPs have a lower capacity to generate heat compared to larger MNPs, primarily due to the impact of sound waves on them. It is worth noting that smaller MNPs have been observed to have enhanced diffusion, allowing them to effectively spread within the tumor. However, their smaller size also leads to rapid elimination from the extracellular space into the bloodstream. To summarize, this study demonstrated that the local injection of MNPs carrying drugs not only enables localized chemotherapy but also enhances the effectiveness of low-intensity ultrasound in inducing tissue thermal necrosis. The findings of this study can serve as a valuable and reliable resource for future research in this field and contribute to the development of personalized medicine.

## Introduction

Cancer is one of the deadly diseases around the world which has extended all over the world and affected many people's lives^1^. The effectiveness of treatment procedure highly depends on the transportation of drug to the tumor, drug performance, and its uptake at the target region. In vivo experiments to investigate the process of drug delivery and drug distribution in tumor sites are usually complex and difficult. Therefore, mathematical modeling was proposed as a novel method to study the drug delivery process to tumor sites^2–4^.

The systemic toxicity occurs in both orally and intravenously delivery of chemotherapy agents resulting in limited amount of drug injected and short duration of drug exposure^5,6^. In this way, local administration of drugs can be helpful to accomplish the full potential of chemotherapy. This method allows us to reach higher drug concentration and lower toxicity in the targeted sites^7^. Also, drug delivery with nanocarriers is an effective way to eliminate the side effects of used drug^8^. Recently, many different therapeutic nanoparticles are developed among which Magnetic Nanoparticles (MNPs) especially possess various biotechnological applications. MNPs are generally well-tolerated in biological systems. This is crucial for medical applications, ensuring minimal toxicity and adverse effects on surrounding tissues. Also, they offer versatility in terms of surface functionalization, allowing for the attachment of various targeting ligands, therapeutic agents, or imaging agents. This facilitates customization for specific applications. MNPs, when appropriately coated, can exhibit enhanced stability and longevity, ensuring sustained therapeutic effects during hyperthermic treatments^9,10^.

MNPs possess the capability to cause tumor ablation by making use of different stimuli fields including ultrasound-induced hyperthermia^11–15^. Ultrasound offers high spatial resolution, allowing for precise targeting of specific regions. This precision is beneficial for focusing heat on the intended area and minimizing damage to surrounding healthy tissues. Furthermore, ultrasound stimulation can influence magnetic nanoparticles to generate localized heat. Ultrasound also can be modulated to selectively activate magnetic nanoparticles in specific tissues. This selectivity helps to avoid unnecessary heating of adjacent healthy tissues and enhances the overall safety of the hyperthermic treatment^10,12,13,16^. Ultrasound is of great importance in diagnostic applications and improving advantageous bio-effects in human tissue, including the penetration of drug to target sites for optimal delivery. In order to increase the drug penetration in tissues and cells, mild or intense hyperthermia is performed to heat body tissues and injected drugs^17^. Low-intensity ultrasound (LIUS) is currently being used for various applications such as drug delivery, cancer therapy, and gene delivery^18^. LIUS has minimal impact on tissue structure and temperature. Therefore, the low intensities used in this method do not cause intense hyperthermia.

**Figure 1:**
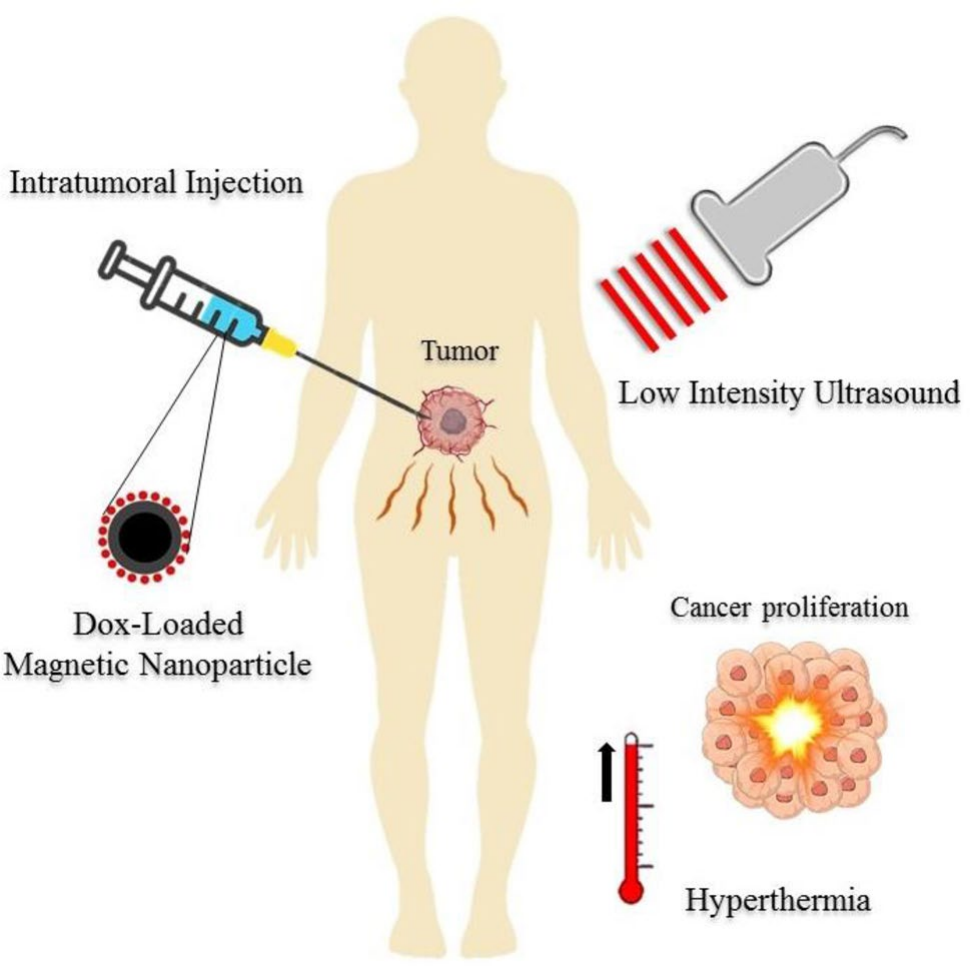
Representation of ultrasound triggered intratumoral drug delivery. Drug-loaded MNPs are administered by intratumoral injection at the tumor site, initiating the controlled release of the drug. Then, LIUS waves are emitted through the tissue, resulting in energy absorption inside the tissue. The energy absorption gradually induces an increase in temperature, leading to thermal ablation.

The use of LIUS has become increasingly popular in diagnostic and therapeutic applications due to its non-invasive nature and minimal side effects such as tumor motion which is frequently seen at high intensities^19^. Although LIUS has offered promising applications for drug delivery and thermal treatments, its inability to significantly raise the tissue temperature has prevented it from achieving the expected potential therapeutic response. Additionally, since ultrasound-triggered hyperthermia using MNPs is a relatively unexplored therapy, a standard protocol for nano-based hyperthermia is not yet available. However, according to the literature, a hyperthermia duration of 30 to 60 min seems to strike a suitable balance for clinical use. This duration allows for an achievable amount of drug delivery thermal ablation, while also considering the technical challenges associated with maintaining controlled hyperthermia and ensuring patient comfort^20–23^. MNPs have an advantage over gold nanoparticles in terms of lower synthesis costs, while their therapeutic efficiency does not show a significant difference^24^. Moreover, when MNPs are exposed to LIUS, in addition to heat generation for therapeutic applications, it also enables treatment monitoring, providing superior capabilities compared to using an alternating magnetic field^25^. Here, a mathematical framework is proposed in this study for the first time with the purpose of using drug-loaded MNPs to increase the efficiency of LIUS. The drug is loaded on the surface of magnetic nanoparticles and released from the nanoparticles at a specific rate according to the characteristics of the tumor microenvironment. Also, the propagation of ultrasound waves can induce hyperthermia, ultimately leading to the thermal necrosis. This study has attempted to present a comprehensive method by taking into account the important governing details, such as fluid flow in the tissue, particle diffusion, acoustic intensity, duration of exposure, and the study period. Figure 1 illustrates an overview of what happens in this research.

## Methods

**Figure 2:**
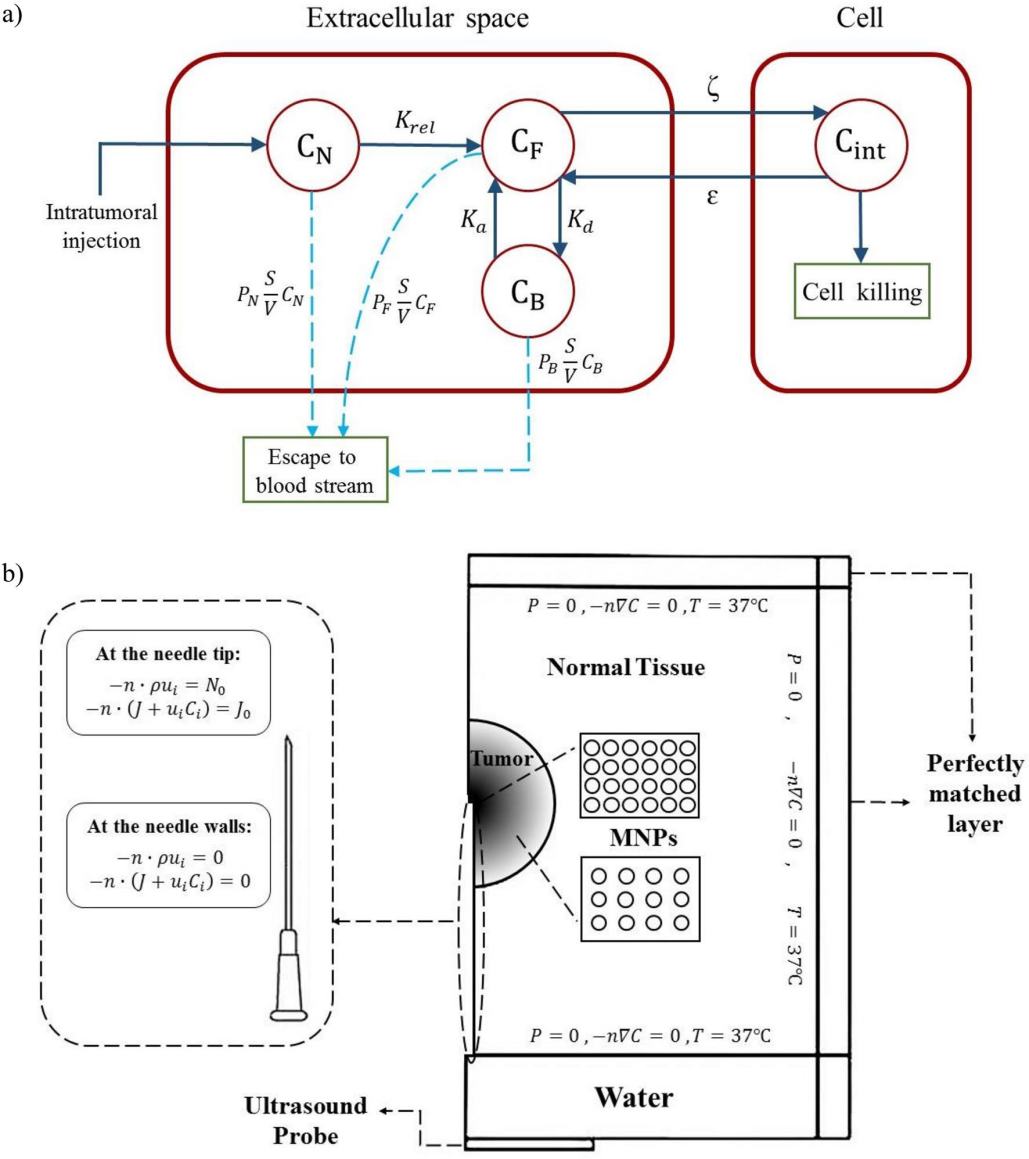
(**a**) Multi-compartment model of the current study which presents the procedure of drug delivery and transport of therapeutic agents : drug loaded MNPs concentration, free drug concentration, bound drug concentration, intracellular drug concentration, drug release rate, association/dissociation rate of drug molecules to protein, ζ, ɛ: cellular uptake and efflux functions. (**b**) Computational domain and boundary conditions implemented in the study are shown. The computational domain contains various components, including a spherical tumor, cylindrical normal tissue, and additional domains relevant to acoustics, such as perfectly matched layers, water, and an ultrasound probe. The distribution of MNPs within the tumor after the injection is shown in the figure. Boundary conditions at the outer boundary of normal tissue, as well as at the injection site, are also represented.

The present study demonstrates the distribution of therapeutic agents in terms of their concentration, taking into account their transportation and biochemical interactions. This is achieved through the utilization of a multi-compartment model (Fig. 2a), which incorporates two key mechanisms: convection and diffusion. The dispersion of therapeutic agents within the extracellular space is described by the convection–diffusion-reaction (CDR) equation. The escape of therapeutic agents from the tissue to the bloodstream is defined by the pore model, which operates through the mechanism of diffusion. Finally, the influx/efflux of therapeutic agents from the extracellular space to tumor cells occurs, ultimately leading to tissue cell death.

Figure 2b presents a schematic representation of the solution domain and boundary conditions. The simulation model incorporates a spherical tumor positioned at the center of a surrounding healthy tissue. The tumor geometry considered in this study consists of a viable tumor with a size of 8 mm, surrounded by a rectangular healthy tissue with dimensions of 36 mm (base) and 24 mm (height). While the specific tumor type is not the focus of this study, it is important to note that the tumor is located in an accessible region, allowing for intratumoral injections, such as in the case of breast solid tumors. The therapeutic agent, doxorubicin, is loaded onto the surface of uncharged Fe_3_O_4_ magnetic nanoparticles, and non-focused low-intensity ultrasound (LIUS) waves are emitted from a linear ultrasound probe.

A comprehensive description of equations, parameters, and their corresponding values is given in the Supplementary File. The subsequent subsection provides an overview of the physics and the governing equations.

- Interstitial fluid flow: Fluid flow in porous media is described by Darcy's equation, which is suitable for interstitial fluid flow in biological tissues. This equation can be used to determine the relationship between interstitial fluid pressure (IFP) and interstitial fluid velocity (IFV), and it can be used for different biological tissues. Therefore, the fluid flow in a tissue can be stated as follows^1,26–28^:
  1 $${v}_{i}=-\frac{\mu }{\kappa }\nabla {p}_{i}$$
- Intratumoral injection of MNPs: Infusion of the nanofluid into tumor site is modeled by applying mass flux condition and concentration flux condition at the needle tip which is governed by the following equations^2^:
  2 $$-n\cdot \rho {u}_{i}={N}_{0}$$
  3 $$-n\cdot \left(J+{u}_{i}{C}_{i}\right)={J}_{0}$$
- MNPs distribution in extracellular space: The distribution of the bare MNPs in the interstitium is illustrated by the convection-diffusion-reaction (CDR) equations. The IFV is computed by solving Darcy’s law, and used in the transient convection diffusion equation for the solute transport as Eq. (4)^2^. Also, the equation for drug loaded MNP is similar to bare MNP except for the drug release term Eq. (5):
  4 $${\kern 1pt}\underbrace{{\frac{\partial {C}_{M}}{\partial t}}}_{MNPs}={\kern 1pt}\underbrace{{\nabla \cdot \left({D}_{eff}\cdot \nabla {C}_{M}\right)}}_{\begin{array}{c}Diffusion \\ mechanism\end{array}}-{\kern 1pt}\underbrace{{\nabla \cdot \left({u}_{i}{C}_{M}\right)}}_{\begin{array}{c}Convection \\ mechanism\end{array}}-{\kern 1pt}\underbrace{{{P}_{M}\frac{S}{V}\left({C}_{N}\right)}}_{Elimination}$$
  5 $$\underbrace{{\frac{\partial {C}_{N}}{\partial t}}}_{\begin{array}{c}Drug Loaded\\ MNPs\end{array}}=\underbrace{{\nabla \cdot \left({D}_{N}\cdot \nabla {C}_{N}\right)}}_{\begin{array}{c}Diffusion \\ mechanism\end{array}}-\underbrace{\nabla \cdot \left({u}_{i}{C}_{N}\right)}_{\begin{array}{c}Convection \\ mechanism\end{array}}-\underbrace{{{k}_{rel}{C}_{N}}}_{Drug release}-\underbrace{{{P}_{N}\frac{S}{V}\left({C}_{N}\right)}}_{Elimination}$$
- Free drug distribution in extracellular space: Transport of the free drug released from MNPs in the interstitium and bound drug which is the drug that bonds to proteins in the interstitium, is governed by the CDR equations^29,30^. Concentration of free drug in the interstitial fluid (Eq. 6) is given as:
  6 $$\underbrace{{\frac{\partial {C}_{F}}{\partial t}}}_{Free Drug}=\underbrace{{D}_{F}{\nabla }^{2}{C}_{F}}_{\begin{array}{c}Diffusion \\ mechanism\end{array}}-\underbrace{{\nabla \cdot \left({C}_{F}{v}_{i}\right)}}_{\begin{array}{c}Convection \\ mechanism\end{array}}+\underbrace{{k}_{rel}{C}_{N}}_{Drug release}-\underbrace{{{P}_{F}\frac{S}{V}\left({C}_{F}\right)}}_{Elimination}+\underbrace{{k}_{d}{C}_{B}-{k}_{a}{C}_{F}}_{\begin{array}{c}\frac{Association}{dissociation}\\ with protein\end{array}}+\underbrace{{D}_{c}\varepsilon -{D}_{C}\zeta }_{\begin{array}{c}\frac{influx}{efflux}\\ from cells\end{array}}$$
- Bound drug distribution in extracellular space: The CDR equation for bound drug is similar to free drug except for the source terms^29^.
  7 $$\underbrace{{\frac{\partial {C}_{B}}{\partial t}}}_{Bound Drug}=\underbrace{{{D}_{B}{\nabla }^{2}{C}_{B}}}_{\begin{array}{c}Diffusion \\ mechanism\end{array}}-\underbrace{{\nabla \cdot \left({C}_{B}{v}_{i}\right)}}_{\begin{array}{c}Convection \\ mechanism\end{array}}-\underbrace{{{k}_{d}{C}_{B}+{k}_{a}{C}_{F}}}_{\begin{array}{c}\frac{Association}{dissociation}\\ with protein\end{array}}-\underbrace{{{P}_{B}\frac{S}{V}\left({C}_{B}\right)}}_{Elimination}$$
- Drug distribution in intracellular space: As only free drug possesses the ability to pass through the cell membrane^31,32^, the cellular uptake depends on the concentration of free drug in the interstitial fluid^29,30^.
  8 $$\frac{\partial {C}_{int}}{\partial t}=\underbrace{{\zeta -\varepsilon }}_{\begin{array}{c}\frac{influx}{efflux}\\ from\, cells\end{array}}$$
- Fraction of survived cells: The change in density of tumor cells with respect to time is calculated using a pharmacodynamics model based on intracellular concentration as described below^33,34^.
  9 $$\underbrace{{\frac{d{D}_{c}}{dt}}}_{Cell density}=-\underbrace{{\frac{{f}_{max}{C}_{int}}{{EC}_{50}+{C}_{int}}{D}_{c}}}_{Anticancer effect}+\underbrace{{{k}_{c}{D}_{c}}}_{Cell proliferation}-\underbrace{{{k}_{g}{D}_{c}^{2}}}_{\begin{array}{c}Physiological \\ degradation\end{array}}$$
- Bioheat transfer: The temperature (T) of tissue resulted from local heating is estimated by solving the energy balance equation^29,35–37^:
  10 $${\rho }_{t}{c}_{t}\frac{\partial {T}_{t}}{\partial t}={{k}_{t}\nabla }^{2}{T}_{t}-\underbrace{{{\rho }_{b}{c}_{b}{w}_{b}\left({T}_{t}-{T}_{b}\right)}}_{\mathrm{Sink term}}+\underbrace{{\left(\underbrace{{{q}_{i}}}_{\begin{array}{c}Wave \\ absorption \end{array}}+\underbrace{{{q}_{v}}}_{\begin{array}{c}Viscous \\ waves\end{array}}+\underbrace{{{q}_{w}}}_{\begin{array}{c}Thermal \\ waves\end{array}}+\underbrace{{{q}_{t}}}_{\begin{array}{c}Temperature\\ gradient \end{array}}\right)}}_{\mathrm{Source term}}$$
- LIUS-mediated hyperthermia: The ultrasound propagation in a tissue is described by linear propagation of the pressure wave equation which is represented by the Helmholtz equation^38^:
  11 $$\frac{1}{\rho {c}_{0}^{2}}\frac{{\partial }^{2}P}{\partial {t}^{2}}+\nabla \cdot \left[-\frac{1}{\rho }\left(\nabla P-{q}_{d}\right)\right]={Q}_{p}$$
- Thermal ablation: The Arrhenius law considers thermally actuated cell damage as a first order irreversible kinetics mechanism in which the cell survival can be predicted using the equation below.
  12 $$\Omega \left(t\right)=ln\left(\frac{C(0)}{C(t)}\right)={\int }_{0}^{t}A{e}^{\frac{-\Delta E}{RT(t)}}dt$$

## Results and discussion

The results of the implemented mathematical model are discussed in this section. First, the IFP and IFV inside the tumor and normal tissue are calculated to determine the convection mechanism on therapeutic agents’ distribution. Additionally, the impact of injection rate on interstitial fluid flow is also investigated. The distribution of therapeutic agents within the tissues is being investigated to assess the impact of acoustic energy absorption and its influence on therapeutic response. Additionally, the impact of key factors such as the size of magnetic nanoparticles (MNPs), the rate of their injection, and drug release rate are evaluated, as these factors have the potential to influence the distribution of therapeutic agents. The subsequent analysis presents the outcomes of ultrasound propagation and the corresponding energy absorption, visualized through acoustic pressure and intensity contours. These findings are then utilized to investigate the increase in tissue temperature and the thermal effects of ultrasound on inducing tumor cell death in the targeted area. This section aims to determine the optimal transducer frequency and power required to initiate hyperthermia, taking into account the biological characteristics of the tissue. Finally, the therapeutic response of the tumor to the combined treatment of chemotherapy and ultrasonic-induced thermal damage is evaluated in the concluding section.

### Fluid flow in the tissue

Tumors typically exhibit an irregular and dysfunctional network of blood microvessels. These abnormal blood microvessels often display heightened permeability, leading to the leakage of fluid and plasma into the interstitial space. Lymphatic networks are responsible for draining excess fluid and waste products from tissues. In tumors, lymphatic vessels may be dysfunctional or insufficient, preventing the efficient removal of interstitial fluid. As a consequence, fluids and other substances accumulate in the tumor tissue, raising the pressure inside it. IFP in both tumor and normal tissues plays a crucial role in drug delivery by hindering penetration. The predicted IFP in the present study for tumor and normal tissues are 1534 Pa and 200 Pa, respectively, which is consistent with experimental studies that have estimated IFP values within the range of 586 to 4200 Pa for tumor tissue and −400 to 800 Pa for normal tissue^39,40^ (Supplementary file, Fig. S2). The results indicate that tumor tissue exhibits a significantly higher value of IFP compared to normal tissue, which displays a much lower IFP. The disparity in IFP between tumor and normal tissue can be attributed to the distinct characteristics of the tumor, such as dysfunctional drainage systems. The pressure gradient at the interface between normal tissue and tumor causes the exchange of interstitial fluid within a narrow region, resulting in an observed increase in velocity in that area.

**Figure 3:**
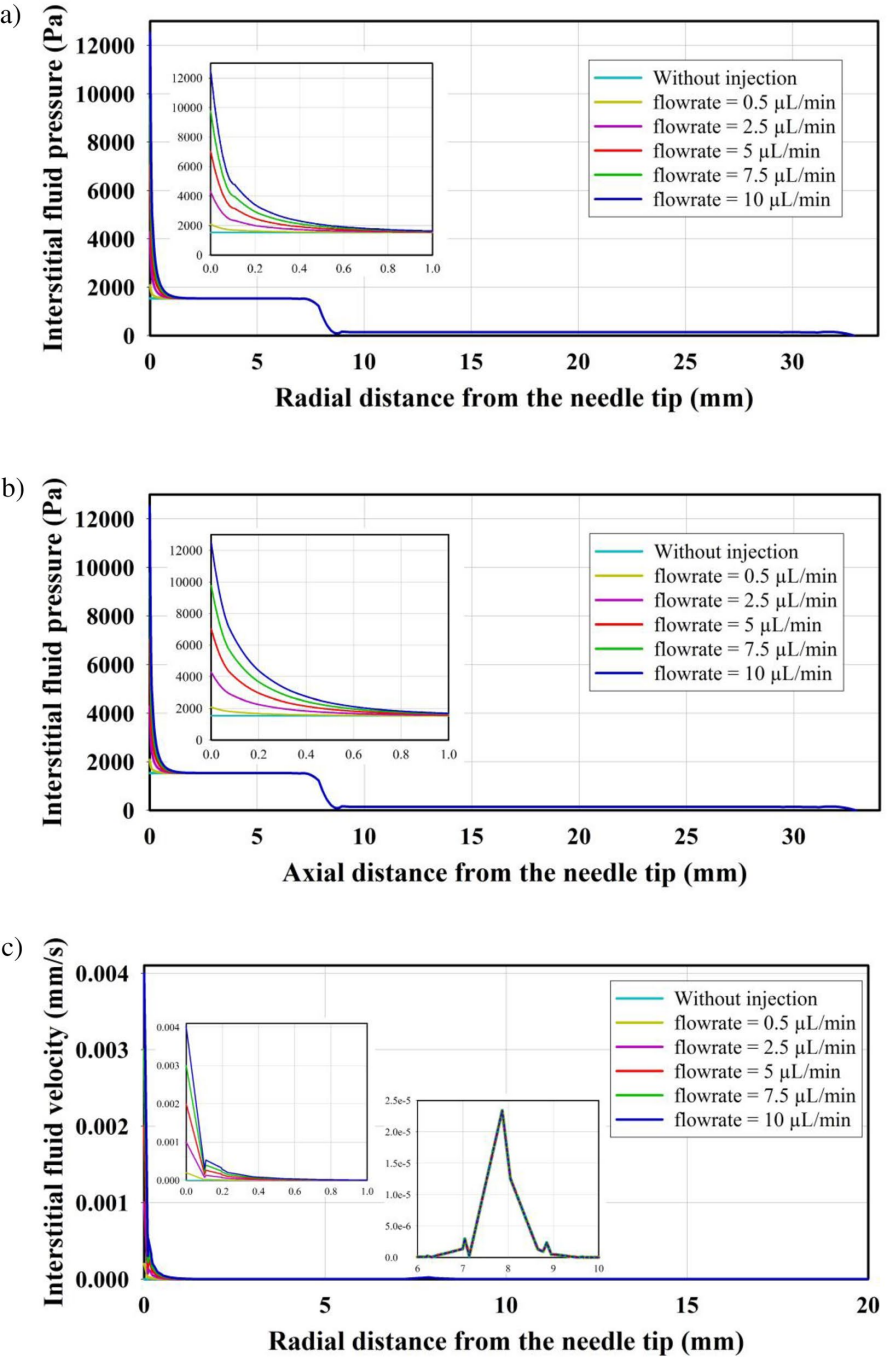

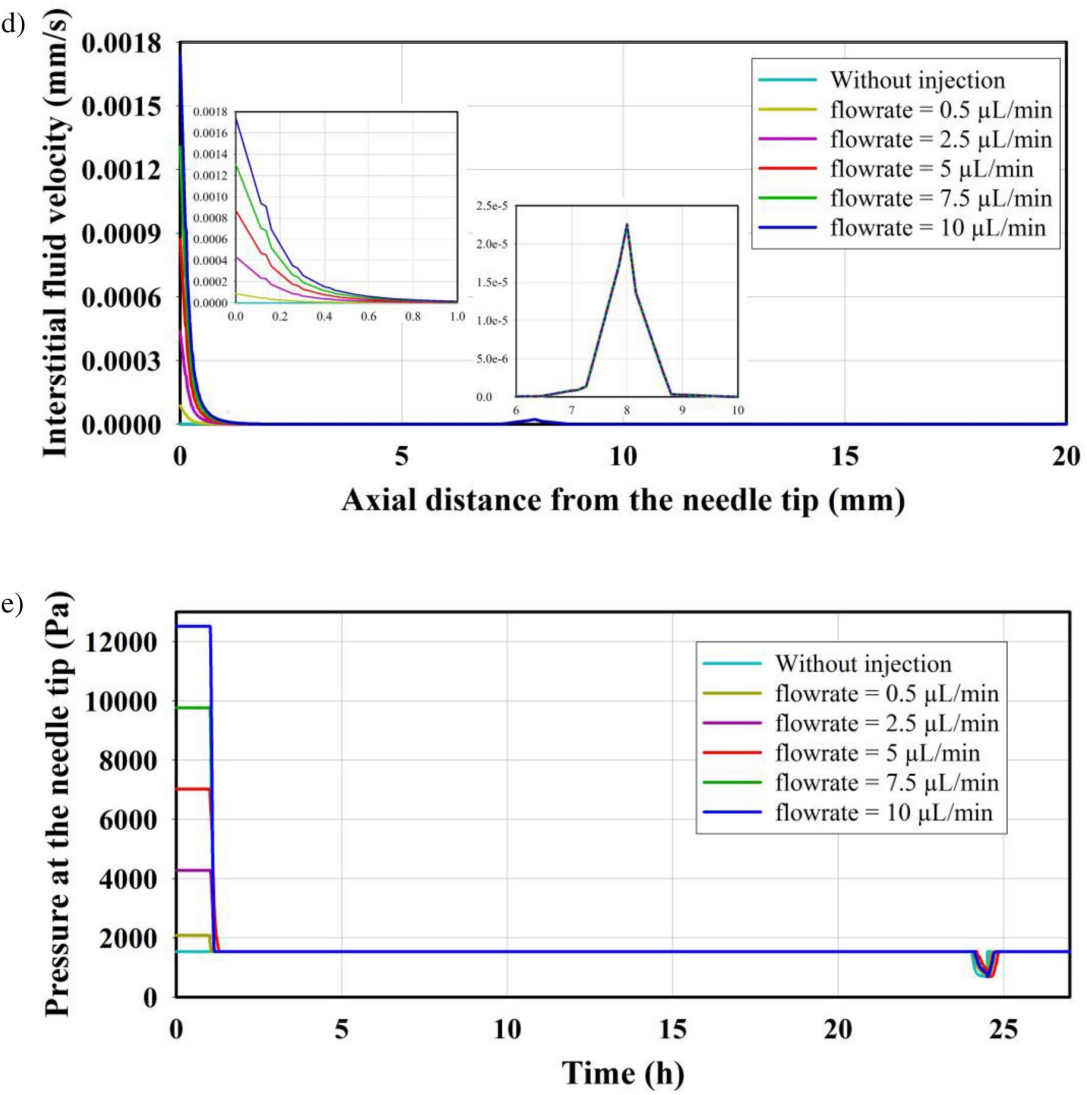
IFP distribution along (**a**) radial direction and (**b**) axial direction, and IFV distribution along (**c**) radial direction and (**d**) axial direction. (**e**) Pressure at the needle tip for different injection rates. Small-scale figures are used to zoom in on specific regions, providing a clear illustration of the variations.

The IFP and IFV profiles are significantly influenced by the flow rate of injected nanoparticles. In Fig. 3, the evaluation involves assessing the gradient of IFP and IFV in both the axial and radial directions of the needle. Except for the needle tip, the predicted pressure and velocity throughout the domain remain the same as without injection case. This indicates that fluid injection only affects a small zone near the needle tip. Considering the results of IFP along the radial direction (Fig. 3a) and the axial direction (Fig. 3b) shows the same maximum pressure at the injection site. The IFP decreases along both directions from the needle tip. It is important to note that in the present study, the drainage system is not considered. The reduction in IFP can be attributed to the entrance of fluid from the interstitium into the bloodstream due to the pressure gradient between the microvessel pressure and IFP, as described in Equation S3. Similar results were observed in the distribution of IFV in both the radial direction (Fig. 3c) and the axial direction (Fig. 3d).

Figure 3e illustrates the predicted pressure in the tumor center and at the needle tip during the entire injection process (t = 0) until the completion of tissue heating (t = 24.5 h). The figure displays the results for six different injection flow rates, considering MNPs with a radius of 20 nm. Simulations show a maximum pressure of 2083 Pa, 4279 Pa, 7024 Pa, 9769 Pa, and 12,514 Pa for injection flow rates of 0.5 , 2.5 , 5 , 7.5 , and 10 , respectively. The decrease in pressure observed during the heating period (t = 24 h to t = 24.5 h) can be attributed to the damage to tumor capillaries caused by the increase in temperature, leading to a cessation of local leakage. However, in the absence of a lymphatic system, IFP increases over time at the area (The thermal effects are further investigated in the subsequent subsections).

### Distribution of MNPs in tumor

The CDR equations are utilized to calculate the spatial–temporal distribution of nanoparticles within the tissues. Five different nanoparticle sizes—5 nm, 10 nm, 20 nm, 50 nm, and 100 nm—are investigated in the present study. To minimize tissue damage, a low injection rate is used, which in turn requires more time to deliver the predetermined volume. However, depending on the therapeutic agent and tumor tissue, the injection time can vary from a few minutes to several days^3,41–44^. Therefore, in this study, the duration of nanofluid injection is set at 1 h. After the injection of nanoparticles, they accumulate in areas near the tip of the needle. Through a combination of convection and diffusion mechanisms, they are able to penetrate distant regions.

**Figure 4:**
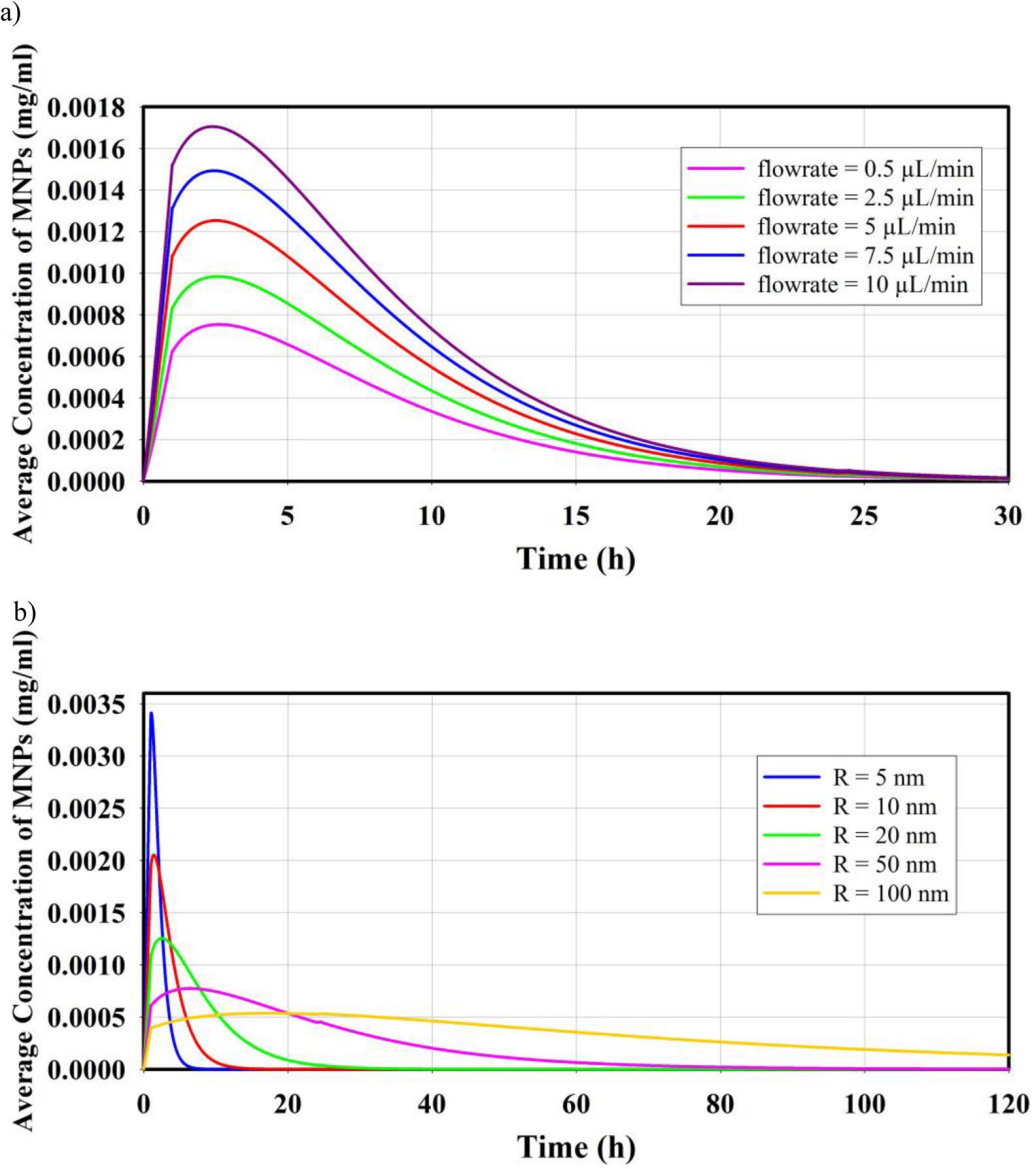
Distribution of MNPs in tumor tissue is shown in figure. (**a**) Effect of injection rate on temporal distribution of MNPs with the size of 20 nm. (**b**) Effect of particle size on temporal distribution of MNPs applying the injection rate of 5.

The rate of injection significantly impacts the average concentration of nanoparticles in the tumor tissue, as demonstrated in Fig. 4a which illustrates the temporal distribution of MNPs with a size of 20 nm. With higher injection rates, a greater quantity of MNPs can penetrate the tumor within the one-hour injection period. Consequently, a larger portion of the tumor will contain MNPs, leading to an increase in the average concentration of nanoparticles within the tumor. Additionally, the findings presented in Fig. 4b highlight the influence of nanoparticle size on the temporal average concentration variation of MNPs within the tumor. The graph demonstrates this impact by comparing the distribution of MNPs for five different particle sizes. Smaller nanoparticles, such as 5 nm MNPs, have the ability to diffuse more quickly into the tumor tissue. This enhanced diffusion enables them to reach farther regions within the tumor and result in higher concentrations. Consequently, the average concentration of 5 nm MNPs is expected to increase. On the other hand, larger MNPs with a size of 100 nm tend to accumulate primarily near the injection region. As a result, the average concentration of these larger MNPs is significantly lower compared to the concentrations observed for smaller nanoparticles. However, the increased permeability of small MNPs allows them to easily enter the blood stream and escape the tumor at a faster rate compared to larger particles. As a result, the concentration of small MNPs decreases more rapidly than that of larger MNPs as depicted in the figure. The maximum concentration of MNPs for particle sizes of 5 nm, 10 nm, 20 nm, 50 nm, and 100 nm is found to be 0.0034, 0.0021, 0.0013, 0.0007, and 0.0005 mg/ml, respectively.

**Figure 5:**
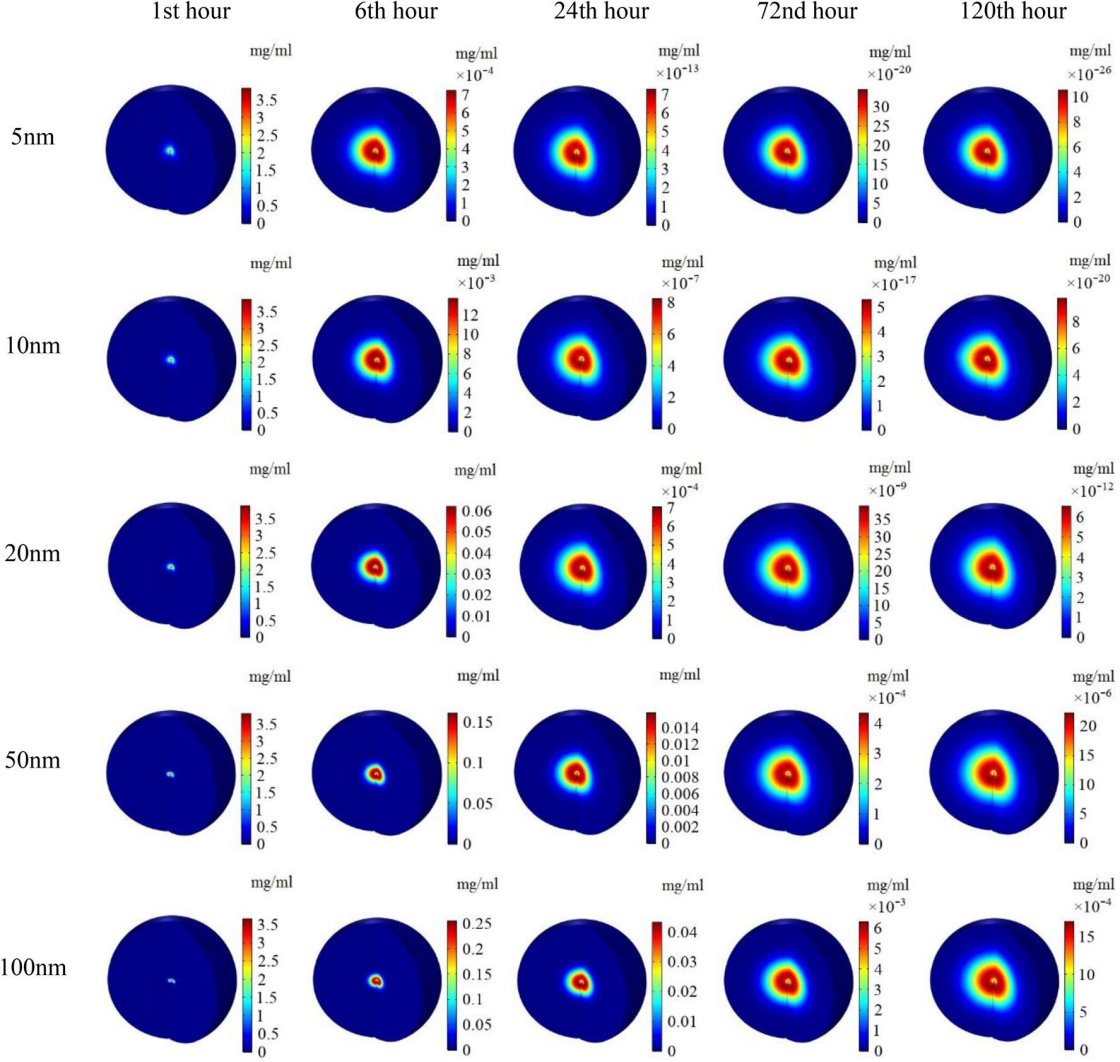
Contours of spatial distribution of MNPs in different times for different MNPs size with the injection rate of 5 $\mathrm{\mu L}/{\text{min}}$.

In Fig. 5, the spatial distribution of MNPs within the tumor is depicted for five different MNPs sizes at various time points. It is observed that, at the last moment of injection, the highest concentration is recorded in the vicinity of the injection site. As time progresses, smaller MNPs are able to penetrate deeper into the tumor, resulting in a significant portion of the tumor being occupied by these MNPs. However, due to the process of removal from the interstitial space and entry into the bloodstream, the concentration of smaller nanoparticles gradually decreases over longer periods of time. On the contrary, larger MNPs, due to their weaker diffusion coefficient, have not been able to reach distant areas like smaller particles even after 24 h of injection. However, due to their slower removal rate from the interstitial space, they exhibit a higher concentration in the tissue for an extended period. After 72 h, the distribution pattern of 100 nm MNPs becomes comparable to that of 5 nm MNPs, but it still maintains a higher concentration within the tissue.

### Distribution of free drug and bound drug

In this study, it is assumed that the drug is loaded onto the surface of MNPs using a linker or incorporated into the coating of the MNPs. Upon injection of the MNPs into the tumor microenvironment, the drug is released in response to specific characteristics of the tumor, such as acidity, for therapeutic purposes. It is further assumed that regardless of the size of the MNPs, the injected concentration carries and delivers the same quantity of the drug. Additionally, the physicochemical properties of the MNPs are assumed to remain constant, resulting in a constant release rate of the drug for MNPs of different sizes. Notably, the released drug, specifically doxorubicin, has a high affinity for binding to existing proteins, including albumin, through a dynamic association/dissociation process in the interstitial space. Therefore, the amount of drug bound to proteins is also taken into consideration in the analysis. Cancer cells uptake the free drug present in the interstitial space of the tumor. This process occurs passively, driven by the concentration gradient between the extracellular and intracellular spaces.

**Figure 6:**
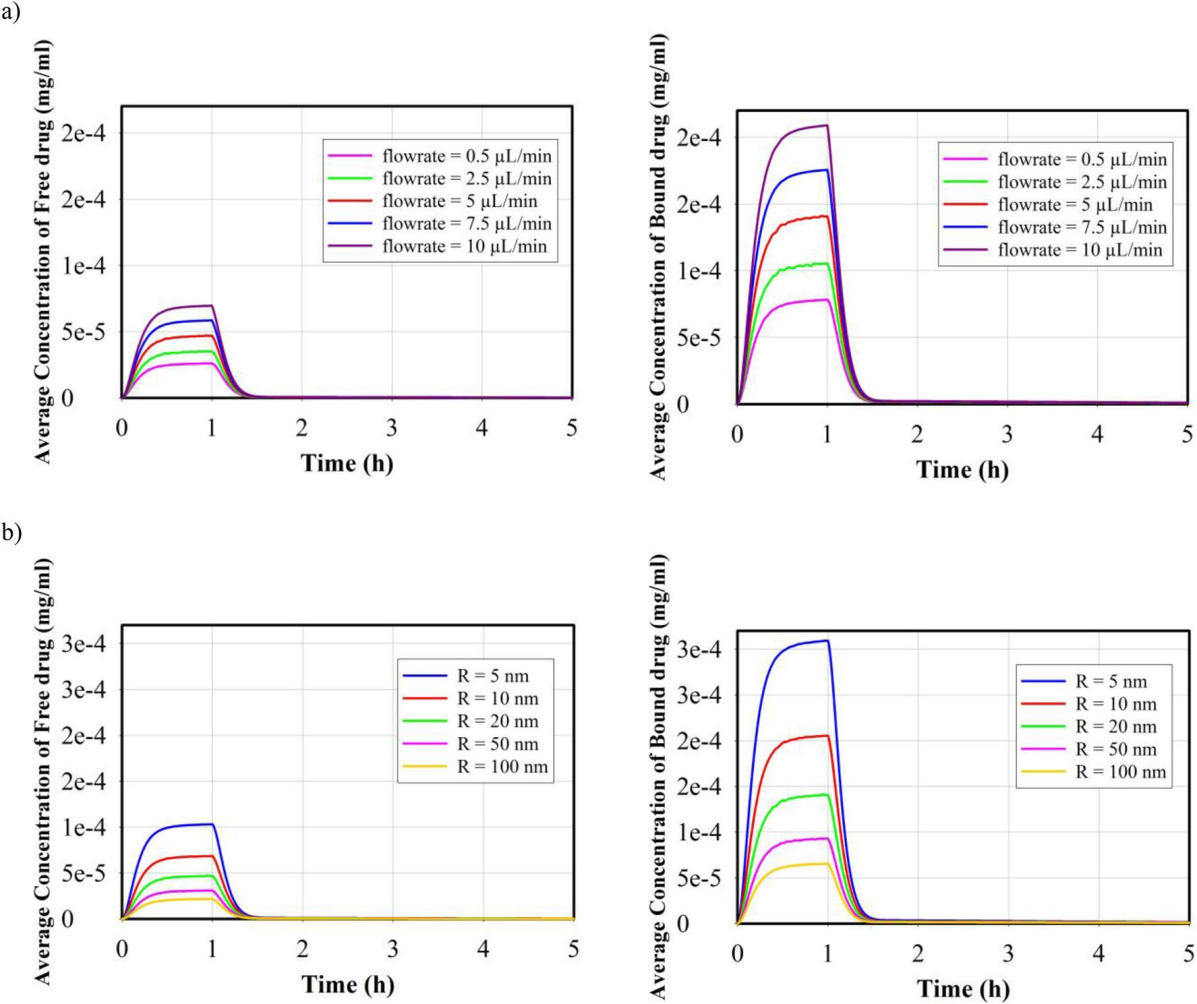
Temporal distribution of free and bound drug; (**a**) temporal distribution of free and bound drug for different injection rates for 20 nm MNPs and release rate of 0.00425 (s^−1^). (**b**) Temporal distribution of free and bound drug for different size of MNPs and injection rate of 5 $\mathrm{\mu L}/{\text{min}}$ and release rate of 0.00425 (s^−1^).

**Figure 7:**
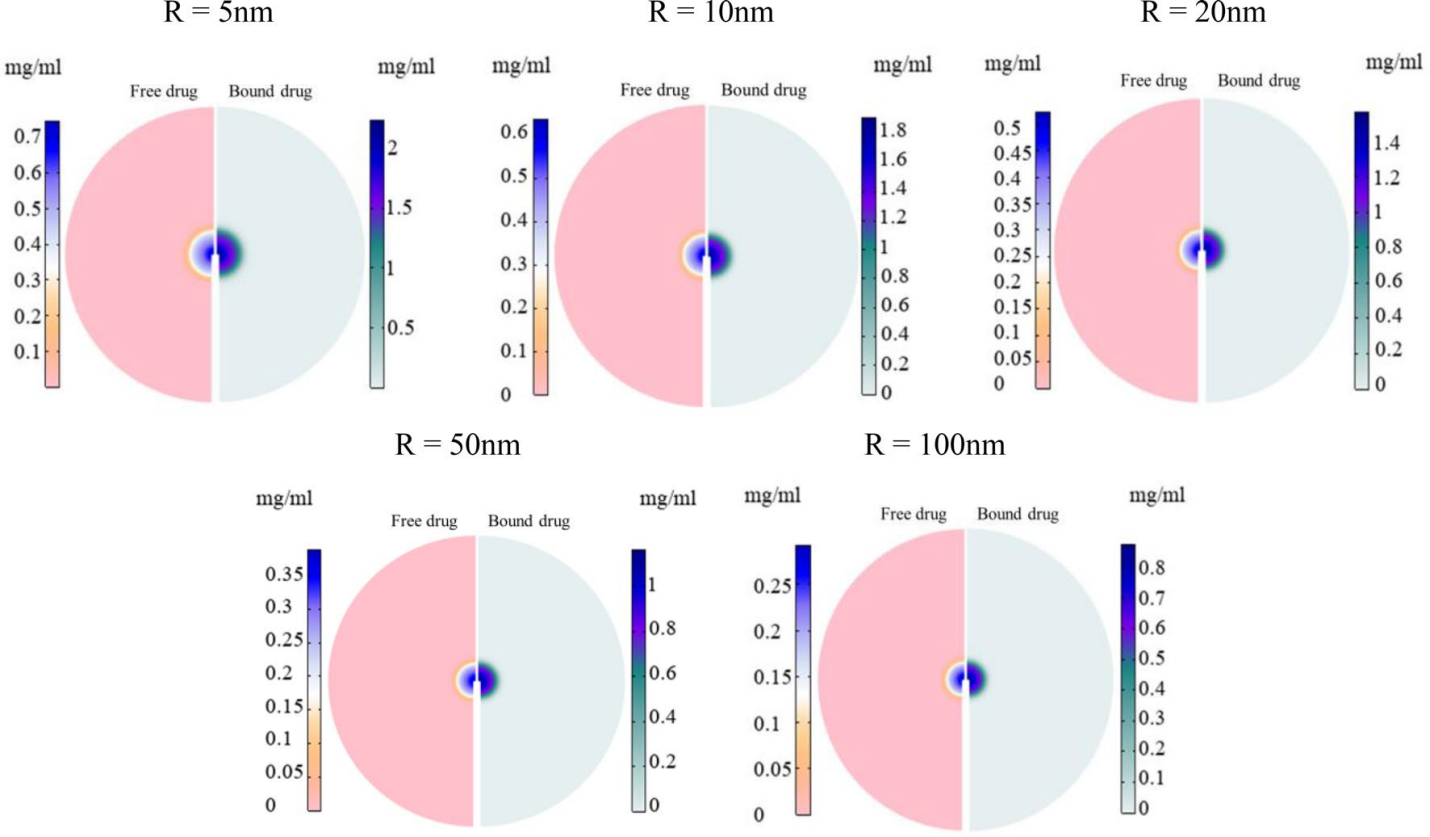
Spatial distribution of free and bound drug in tumor tissue at the end of the injection for different size of MNPs and injection rate of 5 $\mathrm{\mu L}/{\text{min}}$ and release rate of 0.00425 (s^−1^) (the figures are zoomed ×2).

The distribution of free drug and bound drug are shown in Fig. 6. Continuous injection of nanofluid into the tumor can increase the average drug concentration; however, 30 min after the injection ends, the drug concentration reaches zero as a result of the dynamic balance between the source of drug supply (injection) and drug discharge through blood microvessels and internalization by cancer cells. Figure 6a illustrates the concentration of free drug and bound drug in tumor tissue for various injection rates of 20 nm MNPs with a drug release rate of 0.00425 (s^-1^). It is evident that increasing the injection rate leads to an increase in the concentration of drug in the interstitial space of the tumor. For injection rates of 0.5, 2.5, 5, 7.5, and 10 $\mu L/min$, the values of the maximum free drug concentration were calculated as 2.61, 3.51, 4.69, 5.85, and 6.96 ($\times {10}^{-4}{\text{mg}}/{\text{ml}}$), respectively. As depicted in the figure, it is consistent with the literature^45^ that the concentration level of the bound drug is approximately three times higher than that of the free drug. Figure 6b shows the impact of MNPs size for five different MNPs on the temporal distribution of free and bound drugs. As discussed in the previous section, smaller MNPs have the ability to penetrate in a larger area of the tissue and provide a higher average concentration. Furthermore, based on term 3 of Eq. (6), when the release rate remains constant, a higher concentration of small MNPs compared to larger MNPs results in an increased concentration of free drug in the intrastitial space. Consequently, the average concentration of both free and bound drugs in smaller MNPs case is higher compared to their larger counterparts. For MNPs with a size of 5 nm, 10 nm, 20 nm, 50 nm, and 100 nm, the maximum free drug concentration values are 10.3, 6.85, 4.69, 3.1, and 2.18 ($\times {10}^{-4}{\text{mg}}/{\text{ml}}$), respectively. It is also clear that the bound drug has a 3 times higher concentration than the free drug. Figure 7 shows the spatial distribution of free and bound drug inside the tumor at the end of the injection process for different particle sizes. Due to the high rate of permeability, the released drug can easily leave the interstitial space and enter the bloodstream. Also, due to the distribution of smaller MNPs, the drugs released from these MNPs can cover a relatively wider area compared to the drug released from larger MNPs. However, considering the elimination rate (${P}_{F}\frac{S}{V}({C}_{F})$) and the microvascular density depicted in Supplementary File Fig. S1, it is anticipated that the drug will primarily accumulate in areas near the injection site, where the microvascular density is lower.

**Figure 8:**
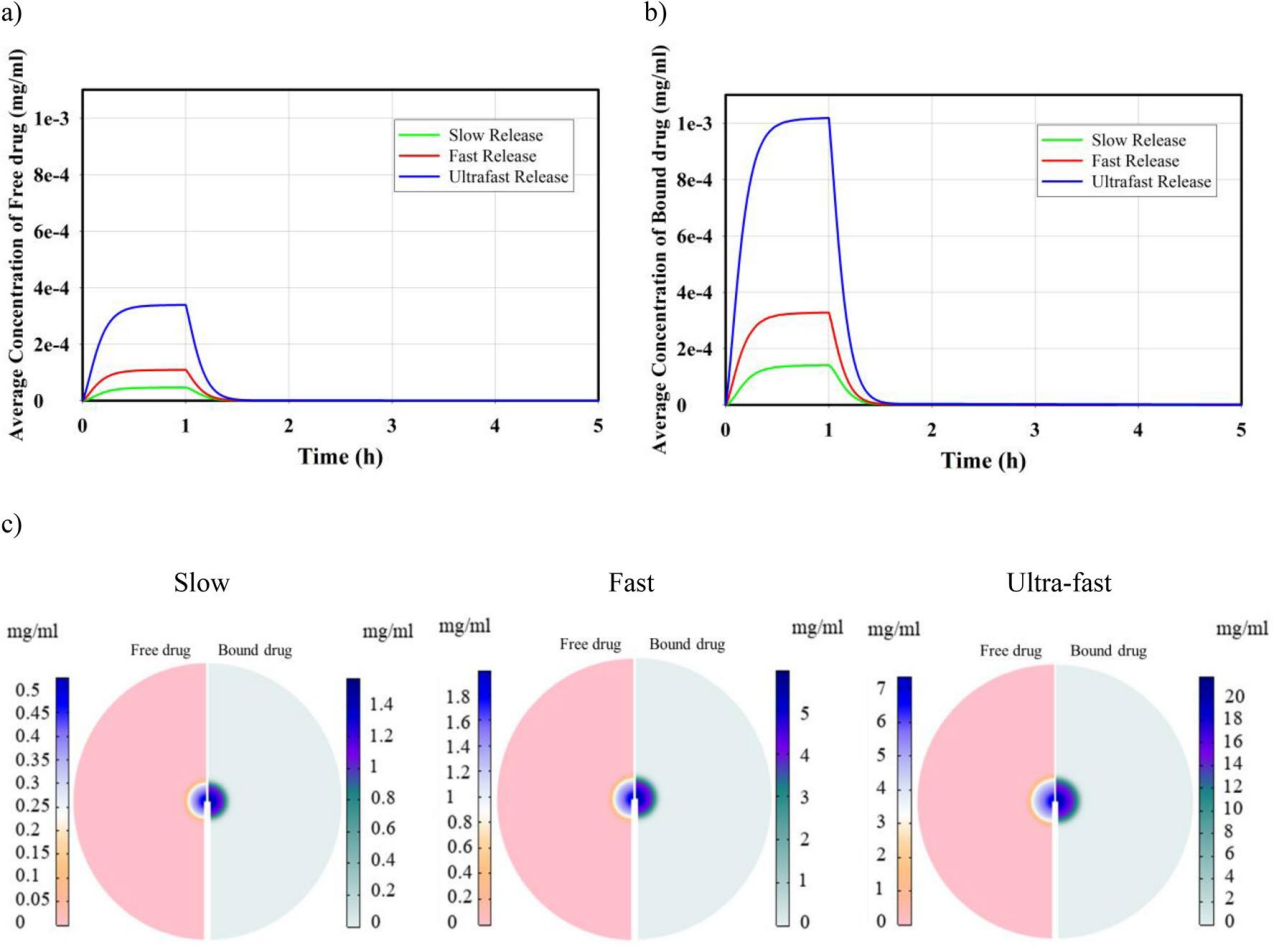
Effect of release rate on (**a**) free drug and (**b**) bound drug temporal distribution for MNPs size of 20nm and injection rate of 5 $\mathrm{\mu L}/{\text{min}}$. (**c**) Spatial distribution of free and bound drug in tumor tissue at the end of the injection for different release rates for MNP size of 20 nm and injection rate of 5 $\mathrm{\mu L}/{\text{min}}$ (the figures are zoomed ×2).

To find the effect of release rate on distribution of drugs, three different drug release rates are chosen, namely slow release (${k}_{rel}= 0.00425 ({{\text{s}}}^{-1})),$ fast release (${k}_{rel}= 0.0425 ({{\text{s}}}^{-1})$), and ultra-fast release (${k}_{rel}= 0.425 ({{\text{s}}}^{-1})$) release rates which are reported in the literature^16^. Considering drug release as a source of drug concentration (Eq. 6), it is anticipated that an increase in the release rate will lead to higher levels of drug concentration. Results in Fig. 8a, b show an increase in concentration of both free and bound drug by increasing the drug release rate from slow rate to ultra-fast release rate. Due to the explosive release of a large volume of drug, the ultra-fast release rate has the highest concentration of released drug compared to the slow and fast release rates. Upon completion of the injection process, the drug-loaded MNPs concentration and released drug cease to increase. Consequently, the concentration of drugs diminishes, and due to the drug's high elimination rate, its concentration declines rapidly.

Figure 8c illustrates the spatial distribution of free and bound drug inside the tumor at the end of the injection process for different release rates. An increased release rate leads to a higher drug concentration in the vicinity of the injection site. Nevertheless, the high concentration of drugs primarily occurs near the injection site where the microvascular density is relatively low.

### Ultrasound triggered hyperthermia

**Figure 9:**
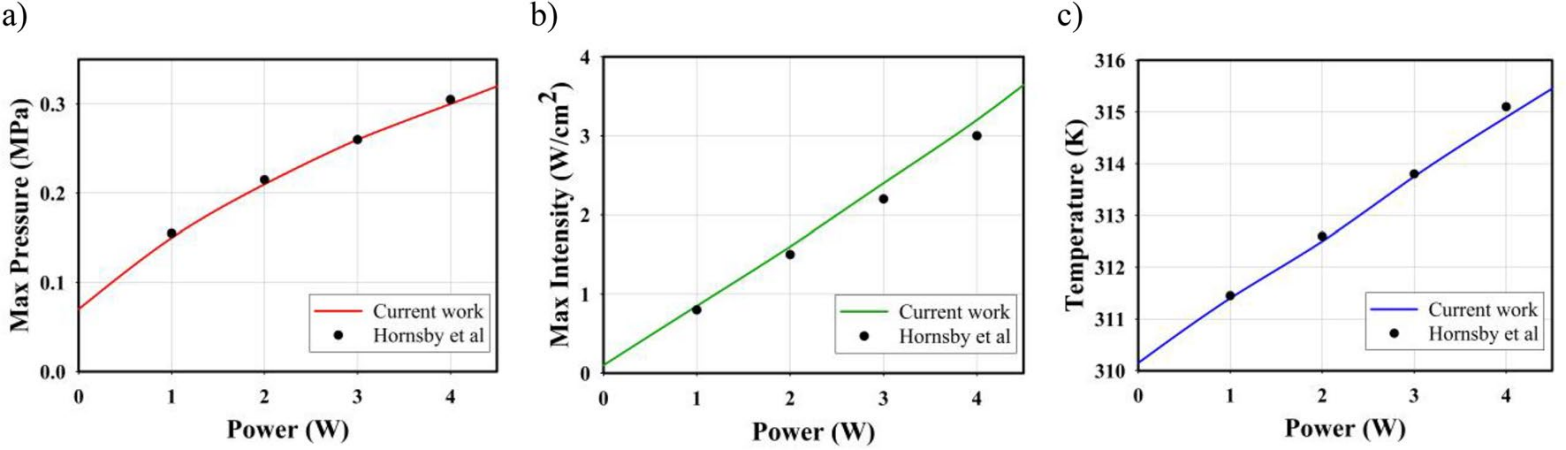
Validation of present study using Hornsby et al.^46^ experimental model. (**a**) Validation of maximum acoustic pressure for a transducer of 1 MHz frequency, (**b**) validation of maximum acoustic intensity for a transducer of 1 MHz frequency, and (**c**) validation of acoustic-triggered average temperature.

The continuous LIUS exposure is simulated in the frequency domain using the Helmholtz equation. Various parameters such as the frequency, power, and radius of the ultrasonic transducer can affect the acoustic pressure and intensity. A study evaluated the LIUS exposure to pig tissue^46^, which is embedded with gold nanoparticles, to estimate the pressure field, acoustic intensity, and resulting temperature. As can be observed, the findings of the present study approximate the results of the referenced paper well (Fig. 9).

**Figure 10:**
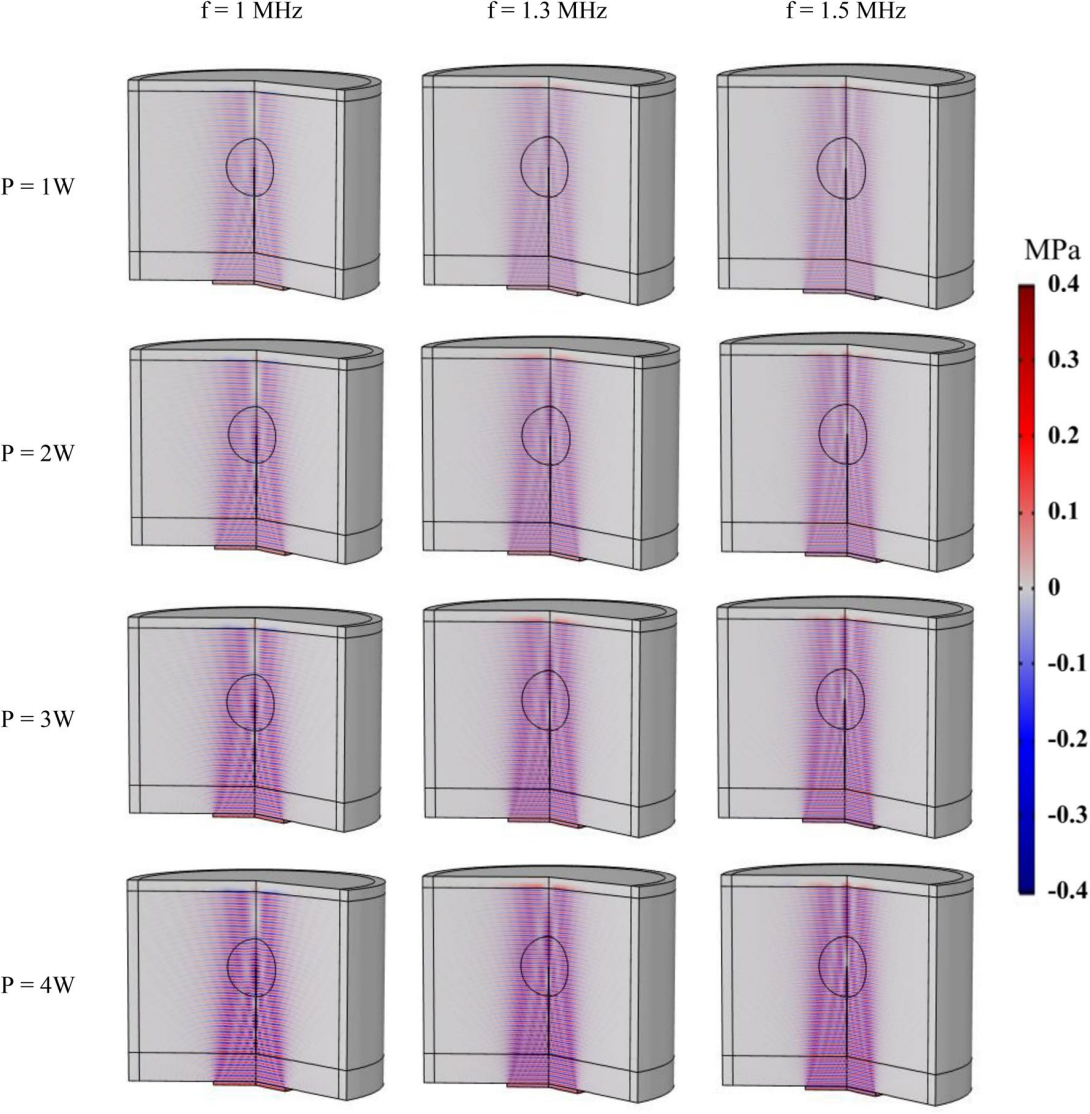
Contours of acoustic pressure for different frequencies and powers of transducer.

**Figure 11:**
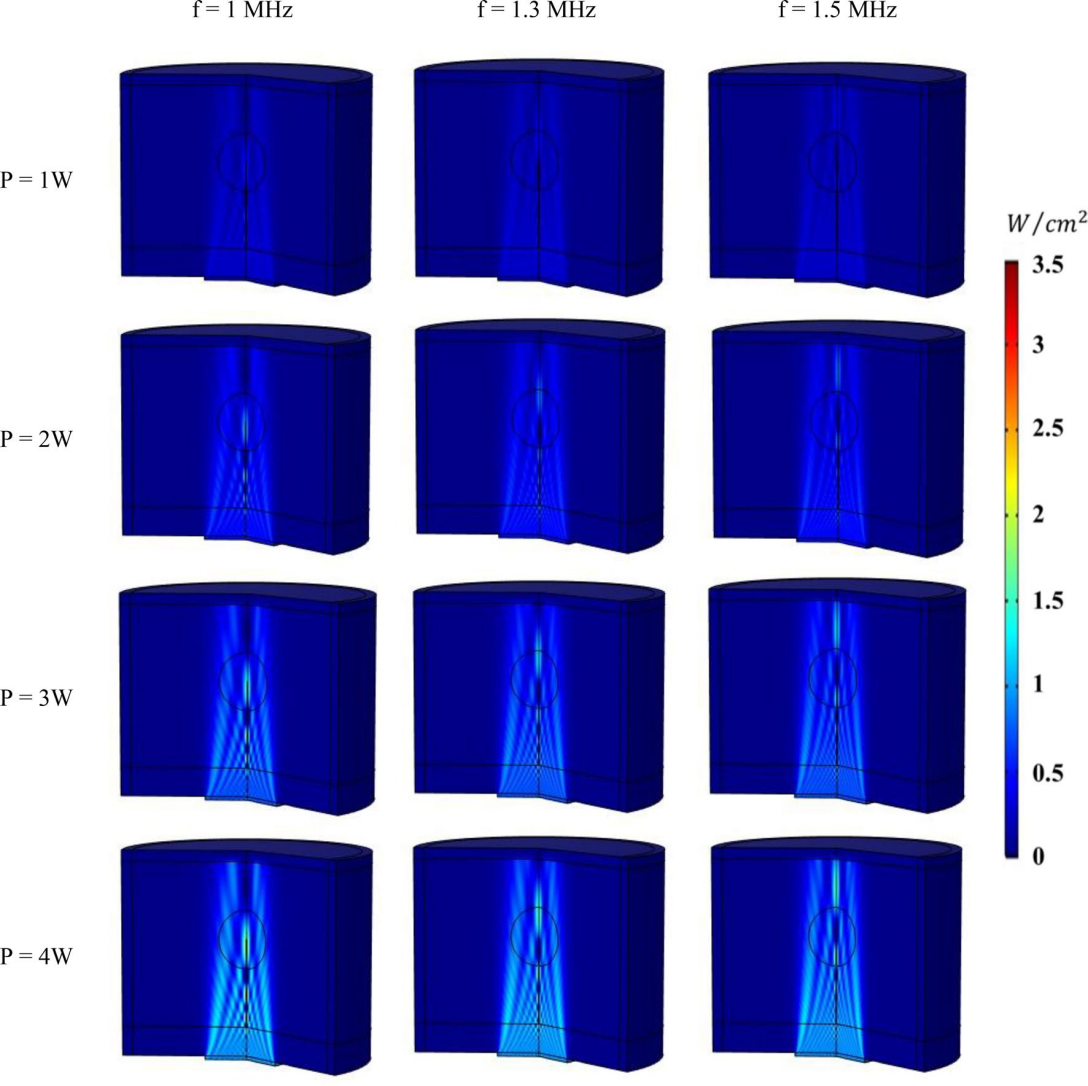
Contours of acoustic intensity for different frequencies and powers of transducer.

The impact of different values of two main parameters, including the and the power of the transducer, on acoustic pressure and intensity is explored in this section. Figures 10 and 11 represent, respectively, the pressure and acoustic intensity contours in the tissue considering three different frequency values (f = 1, 1.3, 1.5 MHz) and four different power values (P = 1, 2, 3, 4 W). Figure 10 clearly demonstrates how changing the frequency of the ultrasonic transducer affects the area that the acoustic waves travel through. In this way, increasing the frequency leads to a smaller area under the cover of ultrasound waves. Additionally, the results of the parametric study shown in Fig. 10 indicate the computed acoustic pressure for all different acoustic power values, demonstrating the direct relationship between the acoustic pressure and transducer power. According to the results, the maximum acoustic pressure for frequency of 1 MHz and power of 1 W, 2 W, 3 W, and 4 W is estimated to be 0.15 MPa, 0.21 MPa, 0.26 MPa, and 0.3 MPa, respectively. Figure 11 displays the measured acoustic intensity contours for different frequencies and power values. This figure shows how changing the frequency of the transducer can affect the location of maximum intensity within the tissue. Furthermore, raising the power of the transducer leads to an increase in acoustic intensity. The acoustic intensity for frequency of 1 MHz and powers 1 W, 2 W, 3 W, and 4 W is estimated to be 0.85, 1.69, 2.54, and 3.38 (W/cm^2^), respectively.

**Figure 12:**
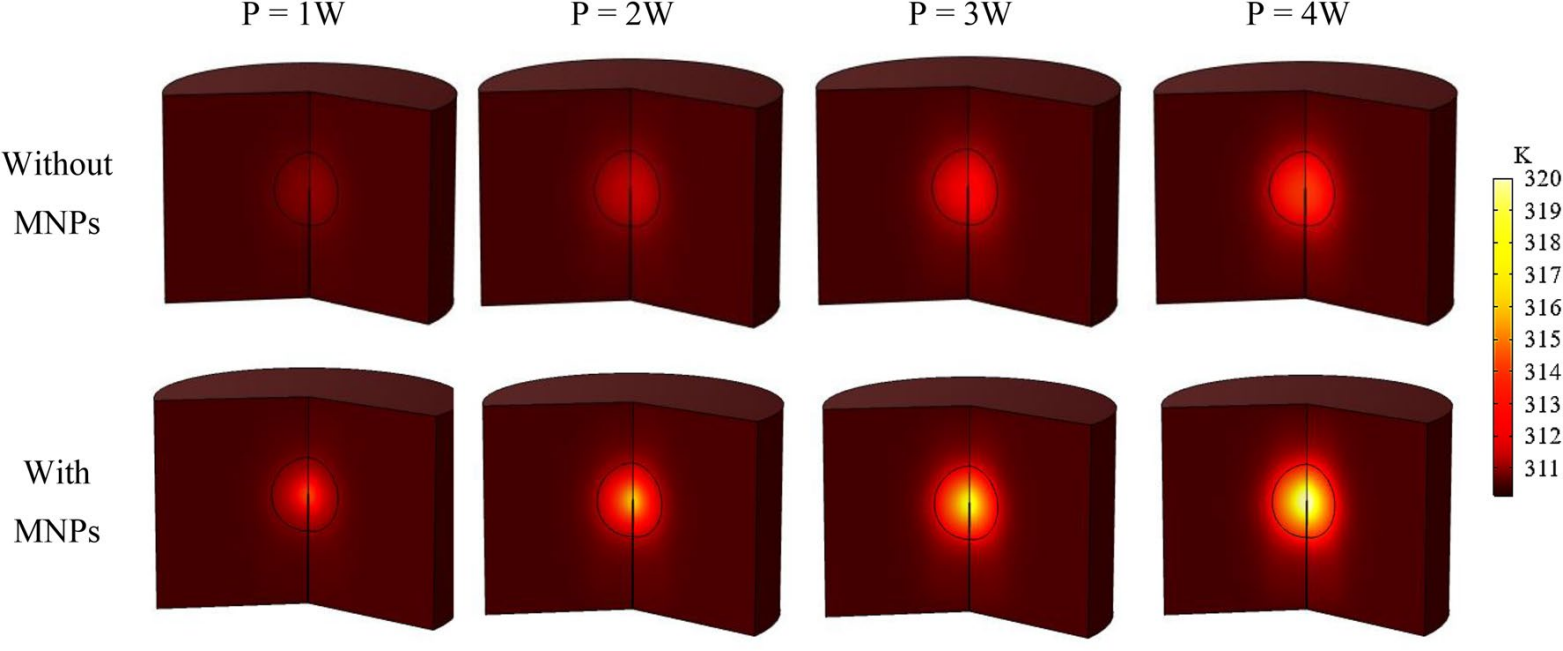
Contours of temperature distribution in tissue as a result of LIUS-triggered heat absorption for different transducer powers and 1 MHz frequency at the end of the exposure (24.5 h) for MNP size of 20 nm.

Figure 12 displays the impact of transducer power on heat generation after 24 h comparing scenarios with and without MNPs injection (20 nm MNPs injection at a rate of 5 $\mathrm{\mu L}/{\text{min}}$). The results indicate that when MNPs are injected, they exhibit a higher heat generation capability compared to the scenario without MNPs injection. This can be attributed to their effective absorption of acoustic energy by MNPs. Lower powers do not have a significant effect on increasing the temperature. However, when the power is 3 or higher, the temperature difference between the cases with and without injection exceeds 5°.

### Therapeutic effects

#### Temperature rise

**Figure 13:**
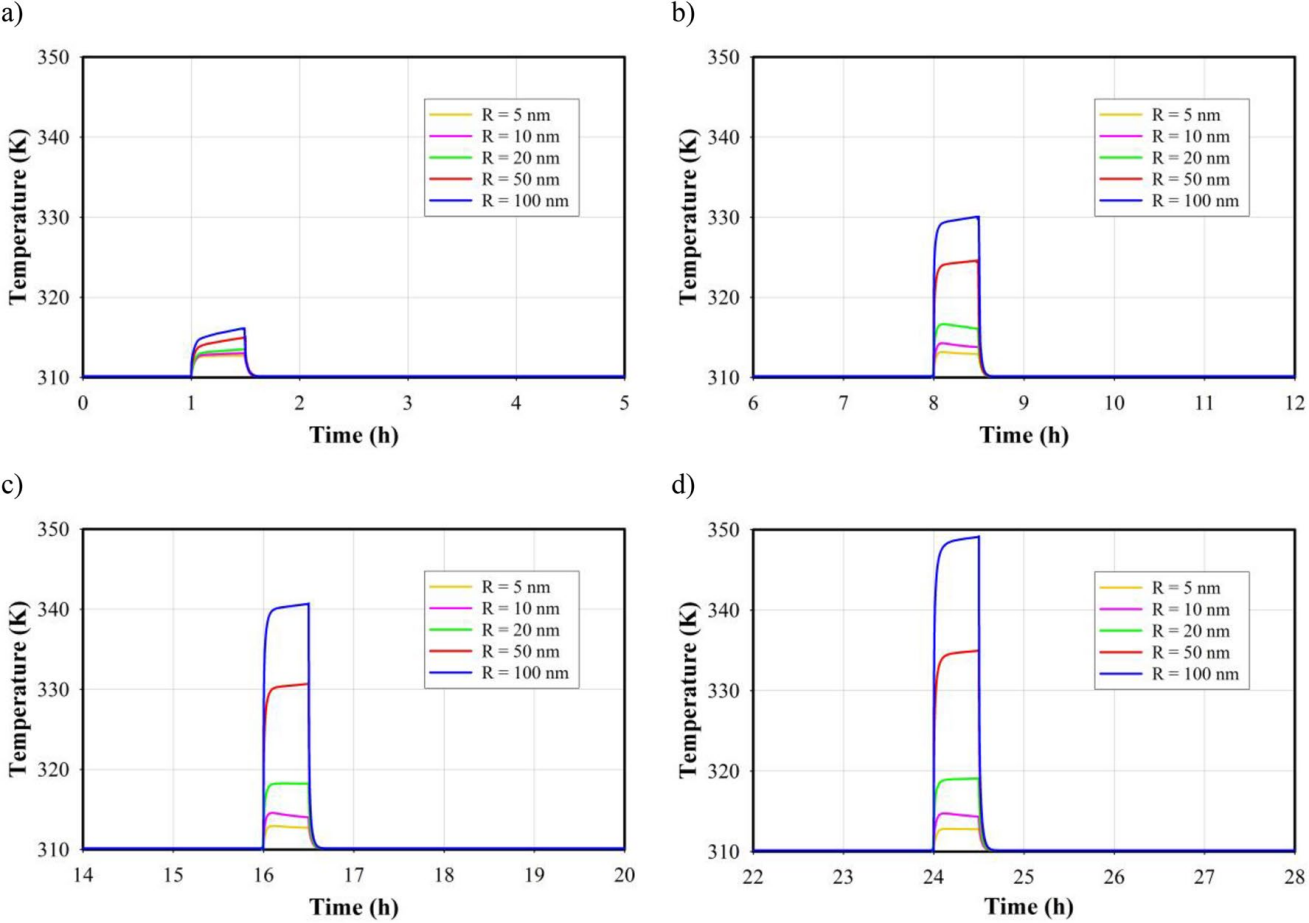
Temporal distribution of maximum temperature in tumor for different particle sizes in different heating times. (**a**) 1st h, (**b**) 8th h, (**c**) 16th h, (**d**) 24th h.

For further analysis in this section, the power and frequency are assumed 3 W and 1 MHz, respectively. The target tissue in the present study is subjected to ultrasound waves for 30 min continually. The tissue temperature rise is influenced by several factors, including the frequency and power of the ultrasound transducer, and presence of MNPs. Figure 13 displays the temporal distribution of the maximum temperature in the tumor tissue for five MNP sizes over different LIUS applying times after injection. It is obvious that the temperature reaches its highest value at the end of the heating process and then rapidly declines once the ultrasound propagation is stopped. According to the results, the temperature increases by shifting the time of applying ultrasound from the first hour to the 24th h. This occurrence arises from the dispersion of MNPs within the tumor after 24 h, resulting in an augmentation of the tissue fraction occupied by nanoparticles. Also, the contribution of thermal mechanisms, including ${q}_{t}$ (Temperature gradient), is subsequently amplified. Based on equations S44, S49, and S50 in the supplementary file, it is evident that the attenuation coefficients of MNPs are dependent on their size. Specifically, increasing the radius of the MNPs results in a higher attenuation coefficient. As a result, larger MNPs have the ability to generate more heat due to their higher attenuation coefficient, leading to higher temperatures compared to smaller MNPs. Interestingly, despite Fig. 4 showing a higher concentration of smaller MNPs, it is observed that larger MNPs, such as those with a size of 100 nm, are able to generate higher temperatures. Also, duo to the thermal conductivity of the tumor tissue, which facilitates the spread of heat throughout the tissue, consequently, the maximum temperature obtained from the use of 100 nm MNPs is higher compared to that obtained from 5 nm MNPs. The greater temperature difference observed in the heat generation between smaller and larger MNPs during the application of acoustic waves after 24 h indicates a lower concentration of smaller MNPs which this lower concentration is attributed to their higher rate of elimination.

**Figure 14:**
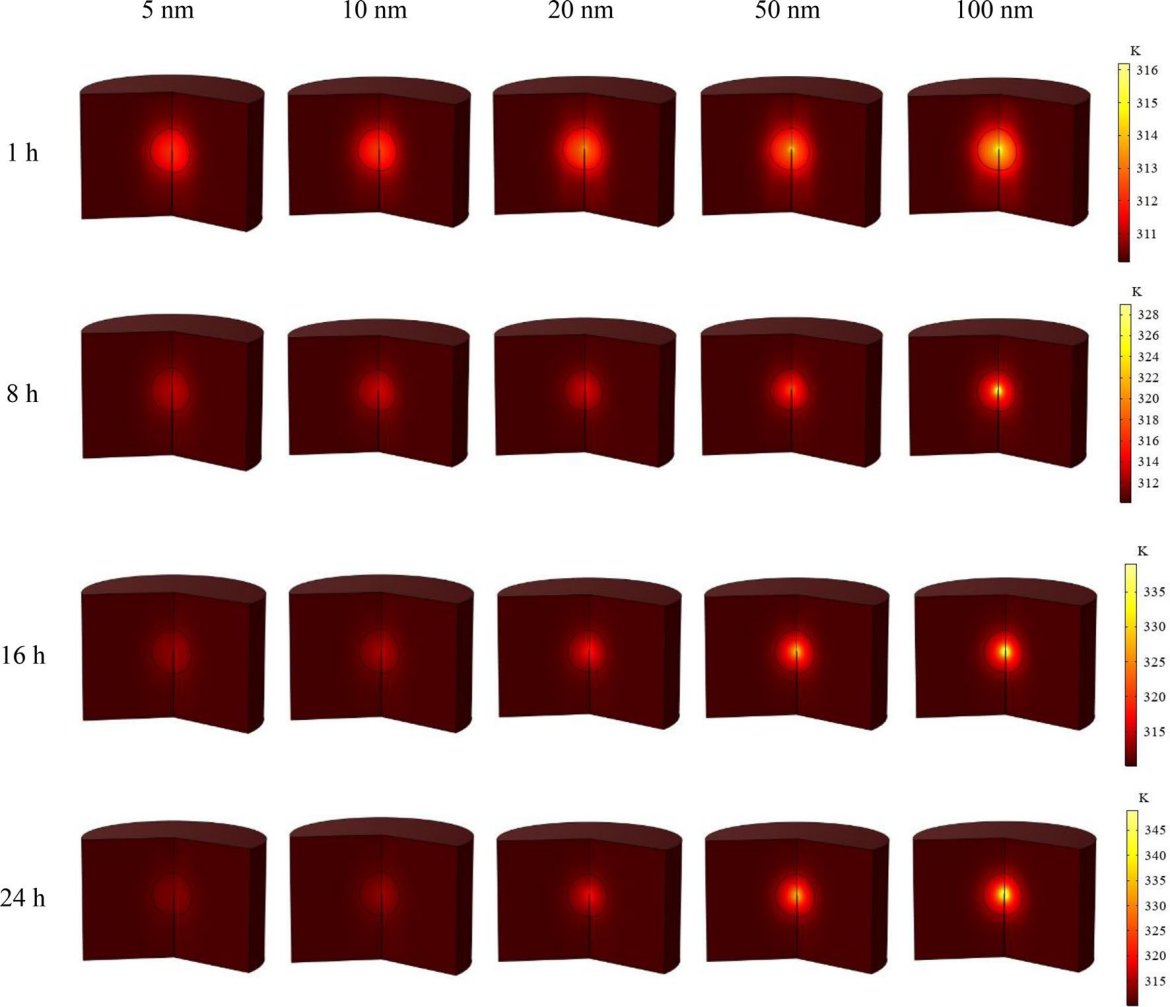
Contours of temperature distribution in tissue as a result of LIUS-triggered heat absorption for different MNP size over different heating times.

Figure 14 illustrates the contours for temperature rise in response to the use of different MNP size over different heating times. The figure shows a positive correlation between the size of MNPs and the temperature values observed specially in the accumulation site. This relationship can be attributed to the larger MNPs' enhanced ability to absorb energy, resulting in higher temperatures. Furthermore, the application of heat at different time intervals, specifically changing from the 1st hour to the 24th hour, leads to an increase in temperature due to the wider distribution of MNPs.

Figure 14 illustrates the contours for temperature rise in response to the use of different MNP size over different heating times. As a general observation, larger MNPs tend to exhibit a greater increase in temperature due to their effective involvement in various mechanisms of temperature generation. During the initial hours, although the concentration of MNPs is high, they occupy only a very small fraction of the tumor. Additionally, the use of un focused LIUS does not have a significant impact on the MNPs within this limited area. Hence, during the initial hours, the absorption of sound waves by the tissue is more efficient, resulting in a mild and non-targeted temperature increase across wider areas. As time progresses, a broader region with a high concentration of MNPs near injection area is achieved. This leads to increased effectiveness in the absorption of waves and other mechanisms of temperature generation, such as temperature gradients and viscous and thermal waves, resulting in a maximum temperature increase in those areas. Simultaneously, as time elapses, MNPs disperse across the tumor's surface, enhancing wave absorption in those specific regions and achieving the desired temperature production. Additionally, the tumor tissue's high coefficient of thermal conductivity contributes to an overall increase in the tissue's temperature. Furthermore, it is also evident that as time progresses, larger MNPs play a more significant role in various mechanisms of temperature production, resulting in a higher increase in temperature compared to smaller MNPs.

#### Probability of tissue damage

**Figure 15:**
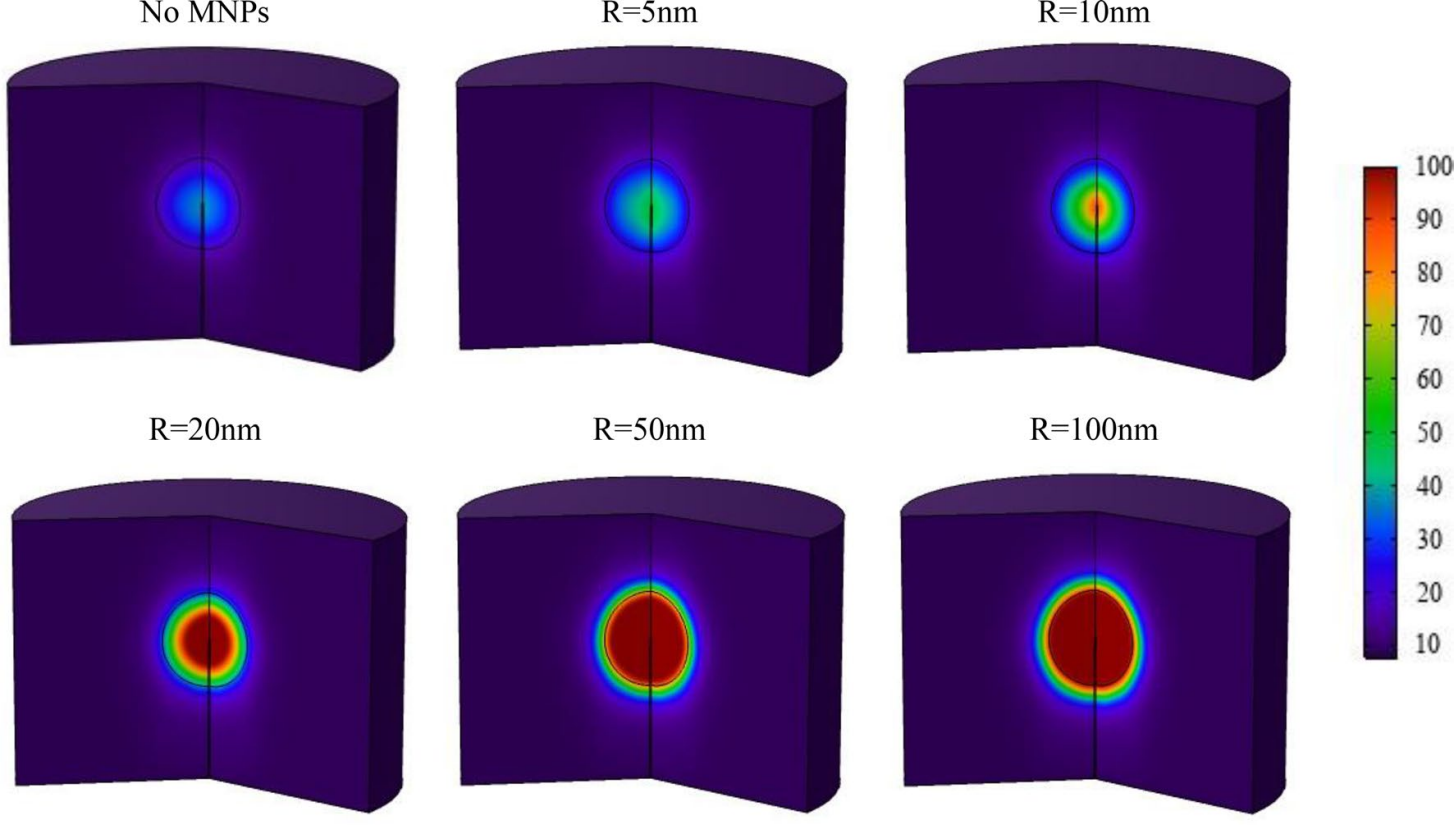
Spatial distribution of probability of tissue cell death as a result of treatment without MNPs and using MNPs with different size at the end of the exposure (24.5 h).

In “Distribution of free drug and bound drug”, it was found that intratumoral injection for drug delivery only covers a limited portion of the tumor tissue, making it insufficient for successful eradication of the tumor. To overcome this problem, thermal ablation can be a promising strategy. For this purpose, the technology of using low-intensity ultrasound waves to obtain hyperthermia is recommended. In this method, 24 h after beginning of the injection process, the tumor tissue is exposed to 30 min of continuous ultrasound propagation at a frequency of 1 MHz and a power of 3 W. As observed in Fig. 14, the temperature rises all over the tumor, yet more thermal energy is generated in the center of the tumor due to the accumulation of nanoparticles and higher absorption of ultrasound waves by them. As a result, higher temperature values and greater probability of cell death are expected in this region. The cytotoxic effect of intratumoral injection on tumor cells is found to be relatively low, in response to the limited area accessible to the drug and the quick clearance of the drug through systemic circulation. Without sufficient drug accumulation in tumor cells, the rate of cell death and physical destruction is lower than the rate of cell proliferation, resulting in an increase in tumor cell density. Therefore, the therapeutic effect of the drug includes a very small part of the treatment process in this study. Figure 15 displays the tumor therapeutic response for five different sizes of MNPs by combining the results of chemotherapy-induced cell death (pharmacodynamic model) and thermal ablation-induced cell death (Arrhenius model). As can be seen, MNPs size and the amount of energy they can absorb are directly related. Consequently, the probability of cell death increases since temperature rises because of an increase in energy absorption. The comparison between cases without MNPs and with the injection of MNPs demonstrates a significant increase in the probability of cancer cell death in the presence of MNPs. The probability of tissue cell death through ultrasound propagation without the use of MNPs is negligible, with a maximum damage probability of 43%. It is evident that there is a very low probability of cell death during the use of nanoparticles with a size of 5 nm and 10 nm for therapeutic purposes. On the other side, nanoparticles with a size of 50 nm and 100 nm can damage a narrow region of healthy tissue around the tumor by 60% and 80%, respectively. Therefore, 20 nm is the optimal size of the MNPs to maximize destruction to the tumor tissue while minimizing damage to the healthy tissue surrounding tumor. It should be noted that due to the absorption of ultrasound energy by the body tissue, about 19% thermal damage to the healthy tissue around the tumor and 7.7% thermal damage to the entire tissue exposed to the ultrasound waves is inevitable.

## Conclusions

The combination of intratumoral injection of therapeutic agents using drug-loaded MNPs and low-intensity ultrasound has shown promise in treating accessible tumors. This approach offers several advantages like the ability to maximize tumor damage while minimizing harm to healthy tissue. The mathematical framework proposed in this study, based on a multi-compartment model, evaluated the effects of ultrasound transducer specifications, nanoparticle size and distribution, and drug release in response to the tumor microenvironment. The results indicate that a higher injection rate increases interstitial fluid pressure and enhances the concentration of the therapeutic agent. Increasing the power and frequency of the ultrasound transducer enhances acoustic pressure and intensity, leading to a greater impact on accumulated MNPs and localized heat generation. However, it was observed that smaller nanoparticles, despite providing a higher average concentration, have a lower acoustic absorption coefficient and therefore cannot generate higher temperatures compared to larger nanoparticles. Overall, this study demonstrates that the local injection of magnetic nanoparticles carrying drugs not only enables localized chemotherapy but also enhances the effectiveness of low-intensity ultrasound in inducing tissue thermal necrosis.

### Supplementary Information

Supplementary Information.

## Data Availability

The datasets used and/or analyzed during the current study are available from the corresponding author on reasonable request.
