# Supplementary material for: Improving tumor treatment through intratumoral injection of drug-loaded magnetic nanoparticles and low-intensity ultrasound
Source: Sci Rep. 2024 Jan 16;14:1452. doi: 10.1038/s41598-024-52003-9 (PMC10791673; doi:10.1038/s41598-024-52003-9)
Supplement: Supplementary file 1 — Supplementary Information. [file 41598_2024_52003_MOESM1_ESM.pdf]

## Supplementary file

# Improving Tumor Treatment Through Intratumoral Injection of Drug-Loaded Magnetic Nanoparticles and Low-Intensity Ultrasound

**Asma Hosseinpour et al.**

This document provides a detailed description of the mathematical model, governing equations and corresponding parameters.

## 1. Governing equations

### 1.1. Interstitial fluid flow

In the first place, the equations representing interstitial fluid flow are solved considering both the tumor and the surrounding healthy tissue to have a porous structure since the drug transport scale is significantly higher compared to the distance between capillaries [1, 2]. Fluid flow in porous media is described by Darcy's equation, which is suitable for interstitial fluid flow in human tissues. This equation can be used to determine the relationship between interstitial fluid pressure (IFP) and interstitial fluid velocity (IFV), and it can be used for different biological tissues. Therefore, the fluid flow in a tissue can be stated as follows [1, 3, 4]:

$$v_i = -\frac{\mu}{\kappa}\nabla p_i \tag{S1}$$

$v_i$ and $p_i$ are IFV and IFP, respectively. $\mu$ and $\kappa$ also indicate the viscosity and hydraulic conductivity of the tissue. To represent the interstitial fluid as an incompressible Newtonian fluid, the mass conservation equation is implemented as given [1]:

$$\nabla u_i = \varphi_V - \varphi_L \tag{S2}$$

where $\varphi_V$ and $\varphi_L$ are the fluid loss rate through the circulatory system and lymphatic vessels, respectively, represented by Starling's law:

$$\varphi_V = \frac{L_p S}{V}\left[p_b - p_i - \sigma_T(\pi_b - \pi_i)\right] \tag{S3}$$

$$\varphi_L = \frac{L_{PL}S_L}{V}(p_b - p_l) \qquad (S4)$$

in which $L_p$ represents the hydraulic conductivity of the blood vessel wall. $S$ refers to the surface area of blood vessels and $V$ stands for the corresponding tissue volume. The blood pressure and interstitial fluid pressure are represented by $p_b$ and $p_i$, respectively. $\sigma_T$ is the averaged osmotic reflection coefficient for proteins in blood. $\pi_b$ and $\pi_i$ are the osmotic pressure of blood and interstitial fluid, respectively. $L_{PL}S_L/V$ demonstrates the effective lymphatic filtration and lymphatic hydrostatic pressure is represented by parameter $p_l$. In the tumor site, fluid drainage by lymphatics is negligible due to lack of functional lymphatic vessels.

**1.2. Intratumoral injection of MNPs**

Infusion of the nanofluid into tumor site is modeled by applying mass flux condition and concentration flux condition at the needle tip which is governed by the following equations [5]:

$$-n \cdot \rho u_i = N_0 \qquad (S5)$$

$$N_0 = \dot{V} \cdot \rho_{nf}/A_{needle} \qquad (S6)$$

in which $\dot{V}$ stands for infusion rate, $A_{needle}$ refers to cross-section area of the needle, and $\rho_{nf} = \varphi\rho_{np} + \rho_{bf}(1-\varphi)$. In this equations, $\varphi$ is volume fraction of MNPs which depends on the radius of the particles, $R$, and obtained from [6]. In addition, $\rho_{np}$ and $\rho_{bf}$ are density of MNPs and base fluid, respectively.

$$-n \cdot (J + u_i C_i) = J_0 \qquad (S7)$$

$$J = D_{eff}\nabla C_i \qquad (S8)$$

$$J_0 = \dot{V} \cdot C_{mf}/A_{needle} \qquad (S9)$$

The term $C_{mf}$ is the concentration of prepared NPs in the base fluid. Intratumoral injection is modeled by considering that the concentration in the injected volume is uniform and equal to the prepared dose.

### 1.3. MNPs distribution in extracellular space: Bare MNPs

The distribution of the nanoparticles in the interstitium is illustrated by the convection-diffusion-reaction (CDR) equations. The IFV is computed by solving Darcy's law, and used in the transient convection diffusion equation for the solute transport as below [5]:

$$\frac{\partial C_M}{\partial t} = \nabla \cdot \left(D_{eff} \cdot \nabla C_M\right) - \nabla \cdot (u_i C_M) + \Phi_M \tag{S10}$$

$$\Phi_M = \Phi_V - \Phi_L \tag{S11}$$

in which, $C_M$ is the concentration of nanoparticles in interstitium and the term $D_{eff}$ accounts for the effective diffusion coefficient. Also, $\Phi_V$ shows the solute's transvascular exchange and the term $\Phi_L$ represents solute's translymphatic transport which are given as:

$$\Phi_V = \varphi_V\left(1 - \sigma_f\right)C_P + P\frac{S}{V}(C_P - C_M)\frac{Pe_M}{e^{Pe_M} - 1} \tag{S12}$$

$$\Phi_L = \varphi_L C_M \tag{S13}$$

$$Pe_M = \frac{F_V\left(1 - \sigma_f\right)}{P\frac{S}{V}} \tag{S14}$$

$$1 - \sigma_f = (1 - \lambda)^2(2 - (1 - \lambda)^2)\left(1 - \frac{2}{3}\lambda^2 - 0.163\lambda^3\right) \tag{S15}$$

$$P = \varepsilon(1 - \lambda)^2 \times (1 - 2.11\lambda + 2.09\lambda^3 - 0.95\lambda^5)\frac{D_0}{\bar{L}} \tag{S16}$$

Where $\sigma_f$ accounts for solute reflection coefficient, $C_P$ is concentration of plasma, which is considered to be zero due to the lack of systemic drug injection, $P$ stands for capillary permeability, and $Pe$ shows the peclet number which is negligible in local drug administration. The term $\lambda = a/r_p$ refers to the ratio of particle size to capillary pore size, in which the parameter a is the radius of solute particle, and $r_p$ is the radius of capillary pore. $\varepsilon$ is the porosity of capillary wall, $D_0$ is related to the free diffusion coefficient, and the term $\bar{L}$ represents capillary wall thickness. The equations mentioned above for calculating the capillary permeability and solute reflection coefficient are taken from a study conducted by Shao et al. [49].

Eventually, considering intratumoral drug injection:

$$\Phi_M = P_M \frac{S}{V} (-C_M) \tag{S17}$$

The MNPs effective diffusion coefficient in the tissue extracellular matrix is defined as solute diffusion in porous medium [7]:

$$\frac{D_{eff}}{D} = \frac{2\varepsilon}{3 - \varepsilon} \tag{S18}$$

$$D = \frac{\kappa_B T}{6\pi\mu a} \tag{S19}$$

in which ε is the void fraction within the tissue space, which is equal to 1 – φ ; $\kappa_B$ shows Boltzmann constant, the term $T$ is the absolute temperature, and $a$ stands for the nanoparticle radius.

**1.4. MNPs distribution in extracellular space: Drug loaded MNPs**

The equation for drug loaded MNP is similar to bare MNP except for the drug release term:

$$\frac{\partial C_N}{\partial t} = \nabla \cdot (D_N \cdot \nabla C_N) - \nabla \cdot (u_i C_N) - k_{rel} C_N + \Phi_N \tag{S20}$$

$$\Phi_N = P_N \frac{S}{V} (-C_N) \tag{S21}$$

where $C_N$ is concentration of MNPs, $D_N$ shows the effective diffusion coefficient of MNPs in the porous media, and $k_{rel}$ is the drug release constant.

**1.5. Drug distribution in extracellular space: Free Drug**

Transport of the free drug released from MNPs in the interstitium and bound drug, which is the drug that bonds to proteins in the interstitium, is governed by the CDR equations [8, 9]. Concentration of free drug in the interstitial fluid ($C_F$) is given as:

$$\frac{\partial C_F}{\partial t} + \nabla \cdot (C_F v_i) = D_F \nabla^2 C_F + k_{rel} C_N + \Phi_F \tag{S22}$$

in which $D_F$ stands for the effective diffusion coefficient of free drug. The term, $\Phi_F$, is the net rate of drug loss to the environment around [8], described by:

$$\Phi_F = P_{fv} + P_u + P_{fb} \tag{S23}$$

where $P_{fv}$, $P_u$, and $P_{fb}$ account for the net rate of drug gained/lost through the blood/lymphatic vessels, cellular influx/efflux from tumor, and association/dissociation with protein, respectively.

$$P_{fv} = P_F \frac{S}{V}(-C_F) \tag{S24}$$

The term $P_F$ is the vasculature walls' permeability for free drug.

The net drug gained/lost related to influx/efflux from tumor cells is governed by:

$$P_u = D_c\varepsilon - D_C\zeta \tag{S25}$$

The term $D_c$ shows the density of tumor cells. ε and ζ are cellular efflux and uptake functions due to multidrug resistance pumps.

Also, the net drug gained/lost caused by protein binding is described by:

$$P_{fb} = k_d C_B - k_a C_F \tag{S26}$$

where $k_d$ and $k_a$ are the dissociation rates and protein binding, respectively.

**1.6. Drug distribution in extracellular space: Bound drug**

The CDR equation for bound drug is similar to free drug except for the source terms [8].

$$\frac{\partial C_B}{\partial t} + \nabla \cdot (C_B v_i) = D_B \nabla^2 C_B + \Phi_B \tag{S27}$$

here $D_B$ is the effective diffusion coefficient of bound drug. The term, $\Phi_B$, is the net rate of drug loss to the environment around [8], given as:

$$\Phi_B = P_{bv} - P_{fb} \tag{S28}$$

where $P_{bv}$ is bound drug gained/lost to the blood/lymphatic vessels which is given as:

$$P_{bv} = P_B \frac{S}{V}(-C_B) \tag{S29}$$

The term $P_B$ is the vasculature walls' permeability for bound drug.

### 1.7. Drug distribution in intracellular space

Only the free drug has the ability to enter cells and when it enters, if it is not removed by circulatory system, it can damage the DNA and destroy cells. As only free drug possesses the ability to pass through the cell membrane [10, 11], the cellular uptake depends on the concentration of free drug in the interstitial fluid [8, 9].

$$\frac{\partial C_{int}}{\partial t} = \zeta - \varepsilon \quad (S30)$$

$$\zeta = V_{max} \frac{C_F}{C_F + k_e \varphi} \quad (S31)$$

$$\varepsilon = V_{max} \frac{C_{int}}{C_{int} + k_i} \quad (S32)$$

here, $V_{max}$ stands for the rate of transmembrane transport, ε and ζ are cellular efflux and uptake functions related to multidrug resistance pumps, constant terms $k_e$ and $k_i$ are obtained from experimental studies, and φ represents the tumor volume fraction.

### 1.8. Fraction of survived cells

The change in density of tumor cells with respect to time is calculated using a pharmacodynamics model based on intracellular concentration as described below [12].

$$\frac{dD_c}{dt} = -\frac{f_{max} C_{int}}{EC_{50} + C_{int}} D_c + k_c D_c - k_g D_c^2 \quad (S33)$$

The first term on the right side of the equation is associated with the anticancer effect, in which $f_{max}$ is constant related to the rate of cell killing and $EC_{50}$ is the concentration of drug generating 50% of $f_{max}$. Also, $k_g$ and $k_c$ are physiological degradation rate and cell proliferation rate constant, respectively. In this paper, physiologic degradation and cell proliferation are considered to have achieved balance at the beginning of each treatment.

### 1.9. Bioheat transfer

For localizing the heating process in treatment of a tumor, the tumor tissue and its surrounding normal tissue are heated. The temperature (T) of tissue resulted from local heating is estimated by solving the energy balance equation [8, 13-15]:

$$\rho_t c_t \frac{\partial T_t}{\partial t} = k_t \nabla^2 T_t - \rho_b c_b w_b (T_t - T_b) + Q_m + Phi \cdot Q_x \tag{S34}$$

The terms $c_t$, $\rho_t$, and $k_t$ represent the specific heat, density, and thermal conductivity of the tissue, respectively. $c_b$, $\rho_b$ and $w_b$ are the specific heat, density, and perfusion rate of blood, respectively. $Q_x$ refers to the produced heat using an external stimulus, which is ultrasound propagation in this study, and $Phi$ is considered as the volume fraction of MNPs distribution in the tissue, which is equal to the ratio of the local concentration to the maximum concentration. $Q_m$ is the heat generated by metabolism and assumed to be zero in present study. In a biological tissue, the heat produced during the NP-assisted ultrasound propagation can diffuse by two different mechanisms: conduction and perfusion. The conduction is described as the transfer of heat from more energetic particles of the medium to less energetic ones due to the interaction between them (the first term on the right side of the equation). On the other hand, the perfusion (the second term on the right side of the equation) is the energy transfer between the medium and blood flow which serves as a sink for the thermal energy generated during LIUS. The rate of heat transfer per unit volume can be defined using an experimental model for perfusion and Fourier's law for conduction [16].

### 1.10. LIUS-mediated hyperthermia

In this study, the ultrasound propagation in a tissue is described by linear propagation of the pressure wave equation which is represented by the Helmholtz equation [17]:

$$\frac{1}{\rho c_0^2} \frac{\partial^2 P}{\partial t^2} + \nabla \cdot \left[ -\frac{1}{\rho} (\nabla P - q_d) \right] = Q_p \tag{S35}$$

here, $\rho$, $c_0$, $Q_p$, and $q_d$ are the density, the speed of sound, possible acoustic monopole and dipole source terms respectively $(Q_p = q_d = 0)$. $Q_p$ is applicable to demonstrate a domain heat source resulting variations of pressure or another nonlinearity in the time domain (Westervelt equation [14]). The term $q_d$ source indicates a domain volumetric force.

Using the Fourier series, the pressure is expanded into its harmonic components:

$$P = psin(\omega t) ==\gg P = pe^{-i\omega t} \tag{S36}$$

By solving the mentioned equation with this complex variable results the Helmholtz equation:

$$\frac{\kappa^2}{\rho}p + \nabla \cdot \left[\frac{1}{\rho}(\nabla p)\right] = 0 \tag{S37}$$

in which κ is the wave number given as:

$$\kappa = {}^{\omega}/_{c} + i\alpha_{ABS} \tag{S38}$$

The term $\alpha_{ABS}$ shows the absorption coefficient. $\omega$ is the angular frequency given by $\omega = 2\pi f$ and $f$ stands for the frequency.

The power ($P_W$) generated by the acoustic waves is a function of the operating parameters of the applied transducer through the $Q_x$ [18]:

$$P_W = 2S\pi^2\rho_w f^2 c_w \Delta x_{max}^2 \tag{S39}$$

here, $S$ is the transducer area, $c_w$ and $\rho_w$ are the speed of sound and the density of the medium which is in touch with the transducer aperture (the water between the tissue and the transducer). The term $\Delta x$ refers to the normal displacement of the transducer aperture which yields to transmittance of the required sound pressure. Accordingly, the transducer power is adjustable by changing the transducer's normal displacement. Coupling the generated pressure field to the desired temperature field is obtained by calculating the deposition of thermal energy achieved by the ultrasonic waves' absorption. Ultrasound waves do not affect the motion of magnetic nanoparticles due to their low intensities.

### 1.11. MNP-enhanced heat transfer during LIUS

The heat produced during ultrasound propagation can be specified by multiple mechanisms described in the following (specified as M1 through M4). The viscous features of the medium that results in the energy dissipation lead the intrinsic wave absorption to happen in a biological tissue. The medium absorbs energy during the propagation of the ultrasound, which is then converted to heat, raising the tissue's temperature. (M1). The presence of nanoparticles and their oscillating motion under acoustic pressure are involved in the other mechanisms. First, layers of viscous waves are created around the NPs and at their interface with the suspending medium as a result of the relative motion of the particles in a medium with viscous properties. The interaction between the viscous and ultrasonic waves generated by the viscous waves affects the attenuation of

ultrasonic waves and results in greater amounts of acoustic energy being absorbed. (M2). Another way that NPs cause temperature changes in the surrounding tissue is by generating a temperature gradient around them as a result of the work that their oscillation under acoustic pressure does. (M3). Additionally, the temperature gradient causes layers of thermal waves to form around the nanoparticles, which interact with ultrasonic waves to increase acoustic energy absorption and, in turn, temperature rise. (M4) [16].

Considering the mentioned mechanisms, during the NP-assisted LIUS propagation, the rate of energy exchange per unit volume is estimated as [16]:

$$q_x = q_i + q_v + q_w + q_t \tag{S40}$$

The first term, $q_x$, relates to the change in density of energy in a control volume during the LIUS propagation. The term $q_i$, represents the heat produced per unit volume as a result of the intrinsic wave absorption process. The terms $q_v$ and $q_t$ describe the heat produced per unit volume as a result of the absorption processes of ultrasonic waves due to the interaction they have with viscous waves and thermal waves, respectively. The term $q_w$ represents the heat produced per unit volume as a result of the temperature gradient around particles.

The rate of heat produced per unit volume as a result of the intrinsic wave absorption process is given as [16]:

$$q_i = 2\alpha_i I \tag{S41}$$

$$I = \frac{p^2}{2\rho c} \tag{S42}$$

The intrinsic absorption coefficient, $\alpha_i$, can be defined as below for a tissue embedded with NPs:

$$\alpha_i = (1 - \varphi)\alpha_a + \varphi\alpha_{NP} \tag{S43}$$

where $\varphi$ represents the volume concentration of NPs. $\alpha_a$ is the intrinsic absorption attenuation coefficient which is obtained from experimental data. $\alpha_{NP}$ is the intrinsic absorption attenuation coefficient of NPs which is estimated by the equation below [19]:

$$\alpha_{NP} = 1.1\frac{\gamma^2\omega^2\tau C_{P_{np}} T_{abs}}{c_{np}^3} \qquad (S44)$$

in which $\gamma$ is the Gruneisen parameter, $\omega$ is the angular frequency of transducer, $C_{P_{np}}$ is the specific heat for NPs, $T_{abs}$ stands for the absolute temperature, and the term $c_{np}$ is the speed of sound for NPs. Also, $\tau$ refers to the phonon relaxation time given by [19]:

$$\frac{1}{\tau} = \frac{1}{\tau_b} + \frac{1}{\tau_{np}} \qquad (S45)$$

$$\tau_b = \frac{3k_{np}}{C_{P_{np}}\rho_{np}c_{np}^2} \qquad (S46)$$

$$\tau_{np} = \frac{d}{c_{np}} \qquad (S47)$$

Parameters $\rho_{np}$, $k_{np}$ and $d$ is density, thermal conductivity, and diameter respectively.

The absorbed acoustic energy at the surface of particles relies on the properties of NPs such as their size and volume concentration, and also on the viscous and thermal properties of both tissue and NPs. By defining the absorption coefficient as $\alpha_{tv}$, for the thermal and viscous mechanisms at the surface of NPs, the energy absorbed per unit volume caused by mentioned mechanisms is described as below [16]:

$$q_{tv} = q_t + q_v = 2\alpha_{tv} I \qquad (S48)$$

For ultrasonic waves in a tissue, $\alpha_{tv}$ can be separated into two parameters as $\alpha_{tv} = \alpha_v + \alpha_t$ , which are the viscous and thermal wave absorption coefficients, respectively.

$$\frac{\alpha_t}{\varphi} = \frac{1}{6}\omega^2 R^2 c_a T_o \rho_a \rho_{np}^2 C_{P\,np}^2 \left(\frac{\gamma_a}{\rho_a C_{P_a}} - \frac{\gamma_{np}}{\rho_{np} C_{P_{np}}}\right)^2 \frac{1}{k_{np}} \left(\frac{1}{5} + \frac{k_{np}}{k_a}\right) \qquad (S49)$$

Here, $R$ refers to the radius of NPs, $T_o$ shows a heat-wave parameter associate with the temperature acquired from Allegra et al. [20].The terms $\gamma_a$ and $\gamma_{np}$ account for the thermal expansion of the tissue and NPs, respectively. Also $k_a$ and $k_{np}$ are thermal conductivity of the tissue and NPs.

$$\frac{\alpha_v}{\varphi} = \frac{18\left(\frac{\omega}{c_a}\right)\left(1-\frac{\rho_a}{\rho_{np}}\right)^2 Y^2(Y+1)}{4Y^4\left(\frac{\rho_a}{\rho_{np}}+2\right)^2 + 36Y^3\left(\frac{\rho_a}{\rho_{np}}+2\right) + 162\left(\frac{\rho_a}{\rho_{np}}\right)^2 Y(Y+1) + 81\left(\frac{\rho_a}{\rho_{np}}\right)^2} \qquad (S50)$$

in which $Y = R[\omega/2v_s]^{1/2}$ , and $v_s$ stands for the kinematic viscosity of tissue.

The temperature gradient produced at the surface of NPs because of the work done by their motion is related to the thermal features of the surrounding tissue, as well as the amplitude of acoustic pressure. Using the heat transfer equation for tissue embedded with solid spherical particles, the heat produced per unit volume caused by the temperature gradient around particles can be represented as below [16]:

$$q_w = T_o \gamma_a (\alpha_v + \alpha_t) c_{mn} p \qquad (S51)$$

where $c_{mn}$ shows the speed of sound in the tissue embedded with NPs described as $c_{mn} = (1-\varphi)c_a + \varphi c_{NP}$ .

### 1.12. Thermal ablation

Thermal ablation process in the tissue was estimated using the Arrhenius law [21]. The Arrhenius law considers thermally actuated cell damage as a first order irreversible kinetics mechanism in which the cell survival can be predicted using the equation below.

$$\Omega(t) = ln\left(\frac{C(0)}{C(t)}\right) = \int_0^t A e^{\frac{-\Delta E}{RT(t)}} dt \qquad (S52)$$

The term $\Omega(t)$, shows the degree of tissue damage, $C(0)$ is the initial value for concentration of healthy cells, $C(t)$ stands for the concentration of healthy cells left over after thermal ablation, $\Delta E$ refers to the activation energy for the thermal stimulation process, $R$ is the universal gas constant, $A$ shows the frequency factor of the kinetic explanation, and $T$ is the absolute temperature during thermal ablation defined as a function of time. The parameters $A$ and $\Delta E$ are dependent on the type of tissue and have been characterized for normal breast tissues by Henriques and Moritz [21] and breast tumor tissues by Bhowmik et al [22]. The possibility of cell death, $P_n(\%)$, is finally described as [23]:

$$P_n(\%) = 100 \times \left(1 - e^{-\Omega(t)}\right) \tag{S53}$$

## 2. Model parameters

All the transport and geometric parameters used in the current work are assumed to be time-independent due to the short evaluation time. The parameters gathered from literature for tissue, drug, MNPs, blood, ultrasound, and drug release are shown in Table1, Table2, Table3, Table4, Table5 and Table6, respectively. Explanations for choosing some of the parameters are given in the following. Since temperature rise due to tissue heating may affect some of the drug transport parameters, these parameters are considered to be temperature-dependent.

### 2.1. Tissue related transport parameters

- Blood vessel surface area to tissue volume ratio

The ratio of blood vessel surface area to tissue volume affects the concentration of anticancer drugs penetrated in the interstitial fluid. It is highly related to the tissue properties and stage of tumor growth [24]. Baxter and Jain [1] recommended using $200\ cm^{-1}$ and $70\ cm^{-1}$ for tumor and normal tissue, respectively. Based on cell proliferation and vascular density, tumors are often divided into three zones; hypoxic, quiescent, and proliferation zone. The hypoxic zone is located in the tumor center due to a low amount of vascular density and poor perfusion. The quiescent zone has moderate perfusion as a result of angiogenesis. Yet in this zone, the vascular density is lower than the proliferation zone. In this regard, a quasi-sinusoidal function is used to specify the distribution of vascular density which is assumed to be zero in the center of the tumor (Figure S1). Moreover, vascular damage and the decline in vascular density due to thermal ablation is considered based on the Arrhenius model (Equation (S52)).

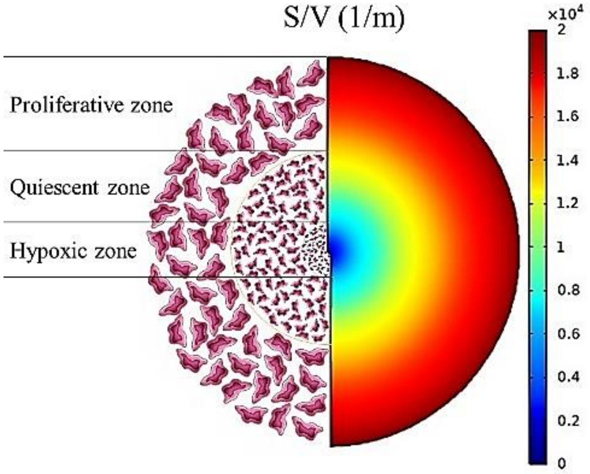


Figure S1. The distribution of microvascular in tumor tissue is non-uniform separating tumor into three regions: proliferation, quiescent, and hypoxia.

- Blood perfusion rate

Blood perfusion distribution is defined as vascular density. Moreover, the rate of blood perfusion ($\omega$) is also measured as a function of temperature [25] as shown:

$$\omega = \omega_0 DS \tag{S54}$$

Where $\omega_0$ is the time dependent blood perfusion at $T = 37^{\circ}C$, and DS has a value between 0 and 1 which represents the degree of vascular stasis [26].

$$DS = e^{-\Omega(t)} \tag{S55}$$

- Thermal conductivity

The dependency of thermal conductivity on temperature is described as given [27]:

$$k(t) = k_0 + 0 \cdot 001161(T - T_0) \tag{S56}$$

In which $k_0$ shows the thermal conductivity at $T_0 = 37^{\circ}C$.

## 2.2. Drug related transport parameters

- Vascular permeability

The vascular permeability coefficient can be used to measure the ability of a microvessel wall to let the transport of substances into and out of the vasculature. The structure of the microvessel wall and the size of the substance are the key factors of effective permeability. [28].

- Free & bound drug

Temperature-dependent drug permeability is defined as a logarithmic function based on a study conducted by Zhan [8]. The permeability for free drug and bound drug is estimated as follows:

$$\frac{P}{P_0} = 10^{0.7(T-T_0)} \tag{S57}$$

Where $P$ and $P_0$ are the permeability of free drug and bound drug at $T$ and $T_0$, respectively.

- MNPs

Based on experimental studies, Zhan [8] defined a relationship between temperature rise and permeability of the nanoparticles given as:

$$\frac{P}{P_0} = -10.54 + 5.76e^{\left(\frac{T-T_0}{5.44}\right)} + 5.78e^{\left(\frac{T-T_0}{5.46}\right)} \tag{S58}$$

Where $P$ and $P_0$ are the permeability of nanoparticles at $T$ and $T_0$, respectively.

- Diffusion coefficient

The diffusion coefficient is often described as the proportion between the molar flux related to molecular diffusion and the negative value of the particle concentration gradient.

- Free & bound drug

The diffusion coefficient is a function of drug's molecular weight [13]. Hence, the diffusion coefficient of free drug and bound drug is not considered as temperature-dependent parameter.

- MNPs

Diffusion coefficient is dependent on viscosity and temperature, according to the Stokes-Einstein equation [8]:

$$\frac{D}{D_0} = \frac{T\mu_0}{T_0\mu} \tag{S59}$$

where $D$ and $D_0$ are the diffusion coefficient of nanoparticles at $T$ and $T_0$, respectively. For this purpose, viscosity of water is considered due to the lack of relevant data. Temperature-dependent viscosity for water is given as below [8]:

$$\mu_w = \exp\left(5.1 - 0.03T + 1.04 \times 10^{-4} T^2\right) \tag{S60}$$

$$\frac{\mu}{\mu_0} = \exp\left(-0.03(T - T_0) + 1.04 \times 10^{-4}(T^2 - T_0^2)\right) \tag{S61}$$

## 3. Solution Strategy

The model including a spherical tumor located in the center of a normal healthy tissue has been simulated in 2D axisymmetric. The tumor geometry considered in this study consists of a viable tumor region of size $R_t$ = 8mm and a healthy tissue around it (in rectangular shape) with base L =36 mm and height H =24 mm. A 3-millimeter-wide perfectly matched layer was applied to the boundaries of the computational domain to prevent LIPUS reflection. The duration of the simulations is significantly shorter than the time needed for tumor growth, and therefore the geometrical and physiological parameters are time-independent. The initial condition for the MNP distribution, free, bound, and intracellular drug concentration is zero all over the domain. Tissue properties at the boundary of tumor and healthy tissue are assumed to be continuous. The nanofluid is considered to be injected directly into the center of tumor via a 26-gauge needle and the needle wall boundary is characterized as a cylindrical surface. At the walls of the needle, a no-flow boundary condition is used $(-n \cdot \rho u_i = 0)$ [5]. Also a no flux boundary condition is considered at the walls of the needle $(-n \cdot (J + u_i C_i) = 0)$. In this study, the injected dose of NPs is considered to be 4 $mg/m^3$ in tumor tissue from the experimental studies [5]. The MNPs are diffused into the tumor region from the injection site (center of the tumor). The size of MNPs is assumed to be 20 nm, so the nanoparticles' distribution in normal tissue is not taken into account as they are larger than the pore size of normal tissue vasculature. A linear ultrasound transducer with specific physical characteristics (Table 5) has been used for thermal ablation process. The location of the transducer is determined in a way that the propagated ultrasonic wave covers the

tumor region. The geometry, mesh, governing equations, initial conditions, and boundary conditions for both tumor and healthy tissue were implemented into a Finite Element Methods software, COMSOL Multiphysics® to acquire numerical solutions. A free triangular meshing was generated throughout the domains. Solution field meshing is considered with a maximum element size of c/f/5 for the section of acoustic streaming where c and f are the speed of sound in tissue and the frequency of ultrasound waves, respectively [29]. To evaluate the effect of mesh element size on the computed results in normal tissue, different mesh sizes were applied. The simulations for normal and tumor tissue were performed using the extra fine element size. For intratumoral injection maximum element size was considered to be 0.05 mm in the injection site. Also the numerical accuracy of the answers is considered to be $10^{-6}$.

| Table 1. Parameters for tissue. | | | | | |
|---|---|---|---|---|---|
| Parameter | Definition | Unit | Tumor | Normal | Reference |
| $\mu$ | Dynamic Viscosity | $Pa.s$ | 0.0078 | 0.0078 | [30] |
| $\rho$ | Density | $kg/m^3$ | 1000 | 1000 | [31] |
| $T$ | Temperature | ℃ | 37 | 37 | - |
| $S/V$ | Surface area of blood vessels per unit tissue volume | $m^{-1}$ | - | 7000 | [3] |
| $K$ | Hydraulic conductivity | $m^2/Pa \cdot s$ | 3.1e–14 | 6.4e-15 | [1] |
| $L_P$ | Hydraulic conductivity of the micro-vascular wall | $m/Pa \cdot s$ | 2.1e–11 | 2.7e–12 | [32] |
| $P_B$ | Vascular fluid pressure | $Pa$ | 2080 | 2100 | [3] |
| $\pi_B$ | Osmotic pressure of the plasma | $Pa$ | 2666 | 2666 | [33] |
| $\pi_i$ | Osmotic pressure of interstitial fluid | $Pa$ | 2000 | 1333 | [33] |
| $\sigma_T$ | Average osmotic reflection coefficient for plasma proteins | - | 0.82 | 0.91 | [1, 34, 35] |
| $a_f$ | Radius of the tumor matrix fibers | $nm$ | 200 | | [36] |
| $r_p$ | Pore radius of vessels | $nm$ | 850 | 10 | [5] |
| $\bar{L}$ | Vessel wall thickness | $\mu m$ | 5 | 5 | [37] |
| $\kappa$ | Permeability of the tissue | $m^2$ | 3.36e-17 | 6.94e-18 | [37] |
| $c_a$ | Speed of sound | $m/s$ | 1550 | 1550 | [38] |
| $C_{P_a}$ | Specific heat | $J/kg \cdot K$ | 3800 | 3600 | [24] |
| $\gamma_a$ | Thermal expansion coefficient | $1/℃$ | 7.323e-5 | 1e-4 | [39, 40] |

| Table 2. Parameters for Doxorubicin. | | | | | |
|---|---|---|---|---|---|
| Parameter | Definition | Unit | Free Doxorubicin | Bound Doxorubicin | Reference |
| $P_{tumor}$ | Permeability of vasculature wall in tumor tissue | $m/s$ | 3.6e-6 | 7.8e-9 | [30] |
| $P_{\mathrm{normal}}$ | Permeability of vasculature wall in normal tissue | $m/s$ | 3.75e-7 | 2.5e-9 | [30] |
| $D_{tumor}$ | Diffusion coefficient in interstitial fluid of tumor | $m^2/s$ | 3.4e-10 | 8.89e-12 | [30] |
| $D_{\mathrm{normal}}$ | Diffusion coefficient in interstitial fluid of normal | $m^2/s$ | 1.58e-10 | 4.17e-12 | [30] |
| $\sigma_d$ | Osmotic reflection coefficient | 1 | 0.15 | 0.82 | [24] |
| $k_a$ | Doxorubicin-protein binding rate | $s^{-1}$ | 0.833 | - | [41] |
| $k_d$ | Doxorubicin-protein dissociation rate | $s^{-1}$ | - | 0.278 | [41] |
| $\varphi$ | Tumor fraction extracellular space | 1 | 0.4 | - | [41] |
| $V_{max}$ | Rate of trans-membrane transport | $kg/10^5 cell \cdot s$ | 4.67e-15 | - | [41] |
| $k_e$ | Michaelis constant for transmembrane transport | $kg/m^3$ | 2.19e-4 | - | [41] |
| $k_i$ | Michaelis constant for transmembrane transport | $kg/10^5 cells$ | 1.37e-12 | - | [41] |
| $f_{max}$ | Cell-kill rate constant | $s^{-1}$ | 1.67e-5 | - | [12] |

| | | | | | |
|---|---|---|---|---|---|
| $EC_{50}$ | Drug concentration producing 50% of $f_{max}$ | $kg/10^5 cells$ | 5e-13 | - | [12] |
| $k_c$ | Cell proliferation rate | $s^{-1}$ | 3e-6 | - | [24] |
| $k_g$ | Cell physiologic degradation rate | $s^{-1}$ | 3e-16 | - | [24] |
| A | Parameter for pharmacokinetic model | $m^{-1}$ | 130 | - | [41] |

Table 3. MNPs parameters

| Parameter | Definition | Unit | Value | Reference |
|---|---|---|---|---|
| $R$ | Radius | $nm$ | 20 | - |
| $\rho$ | Density | $kg/m^3$ | 5170 | [42] |
| $n$ | Number of the MNPs per unit volume | $m^{-3}$ | 1e21 | [43] |
| $\gamma$ | Gruneisen parameter | - | 1.33 | [19] |
| $\kappa_B$ | Boltzmann constant | $J/K$ | 1.38e-23 | [24] |
| $k_{np}$ | Thermal conductivity | $W/m.K$ | 7 | [42] |
| $C_{P_{np}}$ | Specific heat | $J/kg.K$ | 651.26 | [42] |
| $c_{np}$ | Speed of sound | $m/s$ | 4897 | [42] |
| $\gamma_{np}$ | Thermal expansion coefficient | 1/℃ | 11.8e-6 | [19] |

Table 4. Blood parameters

| Parameter | Definition | Unit | Value | Reference |
|---|---|---|---|---|
| $T_b$ | Body temperature | ℃ | 37 | [44] |
| $\rho_b$ | Density | $kg/m^3$ | 1050 | [44] |
| $C_{P_b}$ | Specific heat | $J/kg.K$ | 3500 | [44] |
| $w_b$ | Perfusion rate | $s^{-1}$ | 0.002 (Tumor) 0.018 (Normal) | [45] |

Table 5. LIUS transducer parameters

| Parameter | Definition | Unit | Value | Reference |
|---|---|---|---|---|
| $f$ | Frequency of transducer | $MHz$ | 1 | [46] |
| $P_W$ | Power of transducer | $W$ | 1.4 | [16] |
| $R$ | Radius of transducer | $mm$ | 12 | [29] |

Table 6. Release rates of drug from MNPs [47]

| Release type | Ultra-fast release | Fast release | Slow release |
|---|---|---|---|
| $k_{rel}$ $(1/s)$ | 0.425 | 0.0425 | 0.00425 |

a) b)

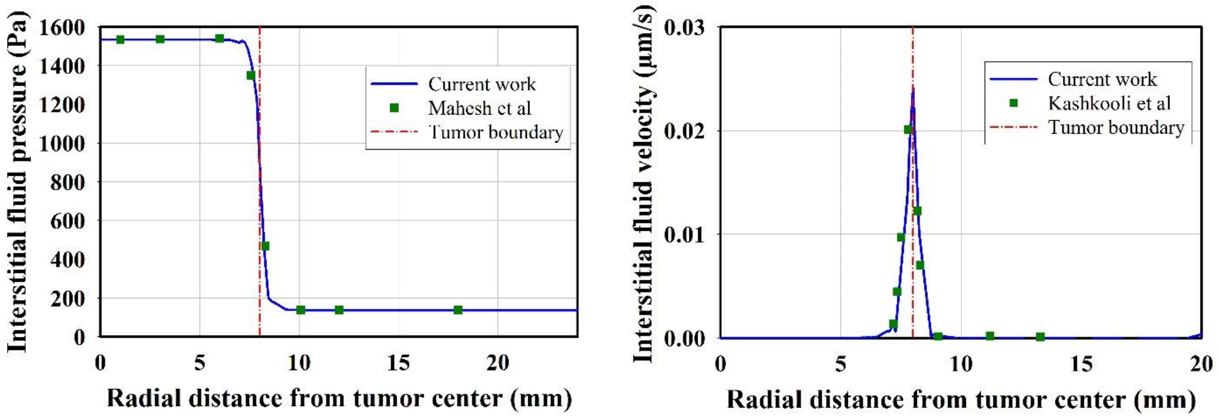


Figure S2. Validation of interstitial fluid flow model with the literature. a) Interstitial fluid pressure is validated using Mahesh et al. [37]. b) Interstitial fluid velocity is also validated using Kashkooli et al. [31].

## References

[1] L.T. Baxter, R.K. Jain, Transport of fluid and macromolecules in tumors. I. Role of interstitial pressure and convection, Microvascular Research, 37 (1989) 77-104.
[2] J.R. Less, T.C. Skalak, E.M. Sevick, R.K. Jain, Microvascular architecture in a mammary carcinoma: branching patterns and vessel dimensions, Cancer Res, 51 (1991) 265-273.
[3] M. Soltani, P. Chen, Numerical modeling of fluid flow in solid tumors, PLoS One, 6 (2011) e20344.
[4] M. Soltani, M. Souri, F. Moradi Kashkooli, Effects of hypoxia and nanocarrier size on pH-responsive nano-delivery system to solid tumors, Scientific Reports, 11 (2021) 19350.
[5] N. Mahesh, N. Singh, P. Talukdar, A mathematical model for understanding nanoparticle biodistribution after intratumoral injection in cancer tumors, Journal of Drug Delivery Science and Technology, 68 (2022) 103048.
[6] M.M. Schmidt, K.D. Wittrup, A modeling analysis of the effects of molecular size and binding affinity on tumor targeting, Mol Cancer Ther, 8 (2009) 2861-2871.
[7] R.L. Fournier, Basic Transport Phenomena in Biomedical Engineering, 2011.
[8] W. Zhan, Mathematical modelling of drug delivery to solid tumour, (2014).
[9] M. Souri, M. Kiani Shahvandi, M. Chiani, F. Moradi Kashkooli, A. Farhangi, M.R. Mehrabi, A. Rahmim, V.M. Savage, M. Soltani, Stimuli-sensitive nano-drug delivery with programmable size changes to enhance accumulation of therapeutic agents in tumors, Drug Delivery, 30 (2023) 2186312.
[10] A.W. El-Kareh, T.W. Secomb, A mathematical model for comparison of bolus injection, continuous infusion, and liposomal delivery of doxorubicin to tumor cells, Neoplasia (New York, NY), 2 (2000) 325.
[11] S. Eikenberry, A tumor cord model for doxorubicin delivery and dose optimization in solid tumors, Theoretical Biology and Medical Modelling, 6 (2009) 16.
[12] R.E. Eliaz, S. Nir, C. Marty, F.C. Szoka, Jr., Determination and modeling of kinetics of cancer cell killing by doxorubicin and doxorubicin encapsulated in targeted liposomes, Cancer Res, 64 (2004) 711-718.
[13] H.H. Pennes, Analysis of tissue and arterial blood temperatures in the resting human forearm. 1948, J Appl Physiol (1985), 85 (1998) 5-34.
[14] M. Rezaeian, A. Sedaghatkish, M. Soltani, Numerical modeling of high-intensity focused ultrasound-mediated intraperitoneal delivery of thermosensitive liposomal doxorubicin for cancer chemotherapy, Drug Delivery, 26 (2019) 898-917.

[15] M. Souri, F. Moradi Kashkooli, M. Soltani, Analysis of magneto-hyperthermia duration in nano-sized drug delivery system to solid tumors using intravascular-triggered thermosensitive-liposome, Pharmaceutical Research, 39 (2022) 753-765.
[16] M. Sadeghi-Goughari, S. Jeon, H.J. Kwon, Analytical and Numerical Model of High Intensity Focused Ultrasound Enhanced With Nanoparticles, IEEE Transactions on Biomedical Engineering, 67 (2020) 3083-3093.
[17] T. Huttunen, M. Malinen, J.P. Kaipio, P.J. White, K. Hynynen, A full-wave Helmholtz model for continuous-wave ultrasound transmission, IEEE Transactions on Ultrasonics, Ferroelectrics, and Frequency Control, 52 (2005) 397-409.
[18] P. Namakshenas, A. Mojra, Numerical study of non-Fourier thermal ablation of benign thyroid tumor by focused ultrasound (FU), Biocybernetics and Biomedical Engineering, (2019).
[19] C. Bera, S.B. Devarakonda, V. Kumar, A.K. Ganguli, R.K. Banerjee, The mechanism of nanoparticle-mediated enhanced energy transfer during high-intensity focused ultrasound sonication, Physical chemistry chemical physics : PCCP, 19 29 (2017) 19075-19082.
[20] J.R. Allegra, S.A. Hawley, Attenuation of Sound in Suspensions and Emulsions: Theory and Experiments, The Journal of the Acoustical Society of America, 51 (1972) 1545-1564.
[21] F. Henriques Jr, A. Moritz, Studies of thermal injury: I. The conduction of heat to and through skin and the temperatures attained therein. A theoretical and an experimental investigation, The American journal of pathology, 23 (1947) 530.
[22] A. Bhowmik, R. Repaka, S.C. Mishra, K. Mitra, Thermal assessment of ablation limit of subsurface tumor during focused ultrasound and laser heating, Journal of Thermal Science and Engineering Applications, 8 (2016).
[23] P.A. Garcia, R.V. Davalos, D. Miklavcic, A numerical investigation of the electric and thermal cell kill distributions in electroporation-based therapies in tissue, PloS one, 9 (2014) e103083.
[24] M. Souri, M. Soltani, F. Moradi Kashkooli, Computational modeling of thermal combination therapies by magneto-ultrasonic heating to enhance drug delivery to solid tumors, Scientific Reports, 11 (2021) 19539.
[25] D.J. Schutt, D. Haemmerich, Effects of variation in perfusion rates and of perfusion models in computational models of radio frequency tumor ablation, Medical physics, 35 (2008) 3462-3470.
[26] W. Zhan, W. Gedroyc, X.Y. Xu, Towards a multiphysics modelling framework for thermosensitive liposomal drug delivery to solid tumour combined with focused ultrasound hyperthermia, Biophysics Reports, 5 (2019) 43-59.
[27] T. Balasubramaniam, H. Bowman, Thermal conductivity and thermal diffusivity of biomaterials: A simultaneous measurement technique, (1977).
[28] F. Yuan, M. Dellian, D. Fukumura, M. Leunig, D.A. Berk, V.P. Torchilin, R.K. Jain, Vascular permeability in a human tumor xenograft: molecular size dependence and cutoff size, Cancer research, 55 (1995) 3752-3756.
[29] T.K. Hornsby, F.M. Kashkooli, A. Jakhmola, M.C. Kolios, J.J. Tavakkoli, Multiphysics Modeling of Low-Intensity Pulsed Ultrasound Induced Chemotherapeutic Drug Release from the Surface of Gold Nanoparticles, Cancers (Basel), 15 (2023).
[30] Y.-M.F. Goh, H.L. Kong, C.-H. Wang, Simulation of the Delivery of Doxorubicin to Hepatoma, Pharmaceutical Research, 18 (2001) 761-770.
[31] F.M. Kashkooli, M. Soltani, M. Rezaeian, C. Meaney, M.-H. Hamedi, M. Kohandel, Effect of vascular normalization on drug delivery to different stages of tumor progression: In-silico analysis, Journal of Drug Delivery Science and Technology, 60 (2020) 101989.
[32] M. Sefidgar, M. Soltani, K. Raahemifar, H. Bazmara, S.M.M. Nayinian, M. Bazargan, Effect of tumor shape, size, and tissue transport properties on drug delivery to solid tumors, Journal of Biological Engineering, 8 (2014) 12.

[33] L.T. Baxter, R.K. Jain, Transport of fluid and macromolecules in tumors: III. Role of binding and metabolism, Microvascular Research, 41 (1991) 5-23.
[34] L.T. Baxter, R.K. Jain, Transport of fluid and macromolecules in tumors. II. Role of heterogeneous perfusion and lymphatics, Microvascular research, 40 (1990) 246-263.
[35] L.T. Baxter, R.K. Jain, Transport of fluid and macromolecules in tumors. IV. A microscopic model of the perivascular distribution, Microvascular research, 41 (1991) 252-272.
[36] A.N. Nacev, Magnetic drug targeting: developing the basics, University of Maryland, College Park2013.
[37] N. Mahesh, N. Singh, P. Talukdar, A mathematical model of intratumoral infusion, particle distribution and heat transfer in cancer tumors: In-silico investigation of magnetic nanoparticle hyperthermia, International Journal of Thermal Sciences, 183 (2023) 107887.
[38] T.W.-H. Sheu, M.A. Solovchuk, A. Chen, M. Thiriet, On an acoustics-thermal-fluid coupling model for the prediction of temperature elevation in liver tumor, International Journal of Heat and Mass Transfer, 54 (2011) 4117-4126.
[39] Y.L. Shao, H.L. Leo, K.J. Chua, Studying of the thermal performance of a hybrid cryo-RFA treatment of a solid tumor, International Journal of Heat and Mass Transfer, 122 (2018) 410-420.
[40] X. Li, Y. Zhong, R. Jazar, A. Subic, Thermal-mechanical deformation modelling of soft tissues for thermal ablation, Biomed Mater Eng, 24 (2014) 2299-2310.
[41] S. Eikenberry, A tumor cord model for doxorubicin delivery and dose optimization in solid tumors, Theor Biol Med Model, 6 (2009) 16.
[42] M. Sadeghi-Goughari, S. Jeon, H.-J. Kwon, Magnetic Nanoparticles-Enhanced Focused Ultrasound Heating: Size Effect, Mechanism, and Performance Analysis, Nanotechnology, 31 (2020).
[43] Q. Wang, Z.S. Deng, J. Liu, Theoretical evaluations of magnetic nanoparticle-enhanced heating on tumor embedded with large blood vessels during hyperthermia, Journal of Nanoparticle Research, 14 (2012) 974.
[44] M. Suleman, S. Riaz, In silico study of hyperthermia treatment of liver cancer using core-shell CoFe2O4@MnFe2O4 magnetic nanoparticles, Journal of Magnetism and Magnetic Materials, 498 (2020) 166143.
[45] P. Vaupel, F. Kallinowski, P. Okunieff, Blood flow, oxygen and nutrient supply, and metabolic microenvironment of human tumors: a review, Cancer Res, 49 (1989) 6449-6465.
[46] T. Hornsby, A. Jakhmola, M.C. Kolios, J.J. Tavakkoli, Significance of Non-Thermal Effects in LIPUS Induced Drug Release from Gold Nanoparticle Drug Carriers, 2021 IEEE UFFC Latin America Ultrasonics Symposium (LAUS), 2021, pp. 1-4.
[47] F. Moradi Kashkooli, M. Souri, J. Tavakkoli, M. C. Kolios, A spatiotemporal computational model of focused ultrasound heat-induced nano-sized drug delivery system in solid tumors, Drug Delivery, 30 (2023) 2219871.
